# Predictors of Food Insecurity and Food Assistance Program Usage Among Puerto Ricans Before and During the COVID-19 Pandemic in Holyoke, Massachusetts

**DOI:** 10.3390/nu16213666

**Published:** 2024-10-28

**Authors:** Destiny Treloar, Dorceta E. Taylor, Ashley Bell

**Affiliations:** Yale School of the Environment, Yale University, 195 Prospect Street, New Haven, CT 06511, USA; dorceta.taylor@yale.edu (D.E.T.); ashley.bell@yale.edu (A.B.)

**Keywords:** soup kitchen, emergency food, immigration, Hispanic, Latino, demographic

## Abstract

Background/Objectives: Puerto Rican households often face elevated rates of food insecurity. Frequently, households experiencing food insecurity turn to federal and emergency nutrition assistance for urgent or prolonged aid. This study analyzes factors influencing food insecurity and food assistance program involvement among Puerto Ricans in Holyoke, Massachusetts, amidst the COVID-19 pandemic. Methods: Using a combination of community-based participatory recruitment, purposive sampling, and web-based tools, we collected 284 survey responses. Our paper employed Pearson’s chi-square, logistic regression, and hierarchical linear models to assess relationships between demographic and household attributes and food access outcomes. Results: Variables such as having children in the household and age significantly influenced food relief participation and food insecurity outcomes. Puerto Rican heritage and low educational attainment were strong predictors of obtaining federal food aid. Low-income participants disproportionately faced food shortages and depended on emergency food initiatives. Our findings indicate that demographic and household factors significantly influence food insecurity and accessibility. Conclusions: Our study demonstrates that the pandemic made it challenging for households to obtain consistent, safe, and affordable nourishment. The impacts were unequally spread; hence, Puerto Rican communities and low-income groups were most affected.

## 1. Introduction

Food insecurity is a public health concern exacerbated by the coronavirus-19 (COVID-19) pandemic [1,2]. The pandemic led to cascading crises and massive disruptions in almost every aspect of the United States food system. For example, the pandemic introduced and exacerbated food system challenges, such as widespread scarcities, skyrocketing food prices, supply chain failures, and meteoric demand for food assistance [3,4,5]. The pandemic also intensified economic difficulties and triggered rising inflation, making it difficult for many to afford groceries or meet their nutritional needs [6,7].

Although the United States is regarded as a “land of plenty” because of its surplus capital and ample food supply, it still faces ubiquitous food insecurity. However, it is not simply a matter of supply; waste is a problem affecting availability. For instance, in 2022, approximately 17 million households were food-insecure, yet a staggering 66.5 million tons of edible food, or roughly 30.0% of the total food supply, were discarded. Therefore, high food insecurity can arise from the amount of food produced, wastage, cost, social inequalities, economic policies, environmental factors, health experiences, and political dynamics [8].

Food insecurity is a complex issue [9,10]. Food access scholarship reveals food security challenges are significantly higher among Hispanic/Latino communities than other groups. (This study will refer to the group being researched as Hispanic/Latino. To delve deeper into this discourse on Hispanic and Latino/a/x/e identities, see [11,12,13,14,15,16,17,18,19,20]). In 2022, approximately 20.8% of Hispanic/Latino households were food insecure [11,12,13,14,15,16,17,18,19,20]. This is nearly double the national average of 12.8% [21].

Food insecurity is inextricably connected to poverty, public health concerns, social mobility, and material hardship [21,22,23,24,25,26,27,28,29,30]. The Brookings Institute reports the Hispanic/Latino population is two times more prone to undergo at least one dimension of poverty than White people [31]. More specifically, food insecurity was more prevalent among Hispanic/Latina single mothers, individuals living in poverty, and urban residents [21,22]. Illnesses are also a confounding element in food insecurity. For example, the Hispanic/Latino population accounted for 28.3% of COVID-19 cases reported nationally [32].

Many studies have underscored the variations in food insecurity within Hispanic/Latino communities. As an illustration, Cordeiro and colleagues noted that Brazilians in Massachusetts experience minimal food insufficiency and express gratitude for the lack of need for food relief [33]. In contrast, Puerto Ricans [33,34,35,36,37,38,39,40,41,42,43,44,45,46,47,48] are susceptible to food access challenges (Puerto Ricans in the United States have a distinct historical, political, and cultural heritage [33,34,35,36,37,38,39,40,41,42,43,44,45,46,47]. In 2021, 5.8 million people of Puerto Rican origin resided in the U.S. [48]). Puerto Ricans report higher food insecurity (25.3%) than other Hispanic/Latino cultural groups [49]. Another study revealed that Puerto Ricans have food insecurity and reduced access to balanced meals compared with Dominicans [50]. Other researchers find that food insecurity differs among Hispanic/Latino groups based on immigration status [51]. 

Scholars have examined the underlying causes of food insecurity in the Puerto Rican diaspora [52,53]. Food insecurity is positively associated with food insecurity and unemployment status, single-mother households, nativity, speaking only Spanish, and limited engagement in Hispanic/Latino cultural activities [54]. Food insecurity also increased when Puerto Ricans did not receive Supplemental Nutrition Assistance Program (SNAP) benefits [55].

Some households cope with unpredictable food availability by relying on food aid [56,57]. The U.S. food relief system is bifurcated. First, the United States Department of Agriculture (USDA) administers 16 nutrition programs [58]. SNAP provides monthly cash benefits and requires non-disabled, childless adults to maintain employment to retain eligibility [59,60]. Women, Infants, and Children (WIC) grants provisions, healthcare services, and dietary instruction to women with lower socioeconomic standing (from pregnancy to post-partum) [61]. Second, many food-insecure individuals resort to emergency assistance programs for free or low-cost food. Emergency food outlets include banks, pantries, soup kitchens, gardens, refrigerators, and drives. The initiatives are often organized and supported by non-profit organizations, corporations, churches, or government agencies [62,63,64,65,66,67,68,69,70,71].

The country’s food assistance safety net comprises government and emergency programs [71,72,73,74,75,76,77] that serve as vital spaces that help alleviate food insecurity in Hispanic/Latino households. According to the USDA, Hispanic/Latino households comprise 21.9% of adult SNAP recipients [21,78], and 49% report reliance on community food programs [79].

As pandemic disruptions persisted, relief networks adjusted, expanded, and intensified their efforts to stave off rising food insufficiency [25,80,81,82]. The federal government responded by introducing economic recovery measures and expanded benefits [83,84,85]. Approximately 11% of the USD 122.1 billion federal budget was directed to creating and supporting USDA nutrition programs as the department revised eligibility criteria and unveiled new programs [86,87,88]. Most notably, SNAP increased financial benefits and offered online purchasing options [7,89,90].

The Federal Families First Coronavirus Response (FFCRA) Act granted the USDA the authority to administer new food insecurity regulations. Consequently, the department raised SNAP benefits to the allowable maximum, authorized supplemental benefits, and relaxed eligibility restrictions [91]. The Pandemic Electronic Benefits Transfer (EBT), which provided maximum benefits for school children, was also introduced [92].

The federal government instituted a plethora of measures to support emergency food networks inundated with food seekers. It expanded The Emergency Food Assistance Program (TEFAP) by channeling resources to local banks and allocating USD 25 billion to food relief networks [93]. TEFAP dispersed over 2.2 billion pounds of food. Other new initiatives included Emergency Meals to You, where non-perishable packages were delivered to youth in rural areas. The Farmers to Families Food Box Program permitted farmers to provide produce, meat, and dairy in boxes to emergency food distributors [94]. Most school districts distributed food through to-go meals. The Summer Food Service Program (SFSP) served students during summer and winter breaks [28,95].

Many community-based programs operated at or beyond capacity because of unprecedented need [96,97]. In 2020, Feeding America reported a 50% increase in participation from 2019 [94]. However, a study found no shifts in food bank usage arising from pandemic conditions in a predominantly African American community in Pittsburgh, Pennsylvania [98].

In 2022, two years after the initial pandemic lockdowns, the government began transitioning from crisis management to adaptation and recovery. As a result, food assistance programs saw a dramatic shift in funding and participation levels. According to Map the Meal Gap’s national study, one in six people used emergency food assistance. That year, the USDA invested USD 183 billion, 6% lower than in 2021, in food security initiatives. Though SNAP and WIC spending increased, Pandemic EBT funding dropped by a sharp 36.9% decrease, bringing the total budget to USD 17.9 billion.

In 2022, the level of participation in federal nutrition initiatives shifted. On average, 41.2 million individuals (12.4%) utilized SNAP monthly benefits, 0.8% lower than in 2021. There was no change in WIC participation, where 6.3 million people participated, the same participation rate as in 2021. NSLP, SBP, and SDSP served a combined 9.4 billion meals. The nutrition programs focusing on children were also mandated to comply with pre-pandemic regulations. The SNAP Online Purchasing Pilot continued to permit recipients to utilize their benefits at eligible online retailers. These expansions, reforms, and reductions reflect the changing dynamics of food assistance needs as the pandemic waned. Although significant strides were made to adapt to the soaring demand for food relief, these efforts encountered inconsistent implementation, disrupted disbursement, and logistical and administrative challenges [28,95].

This study examines the extent of food security experiences and enrollment in government and community aid initiatives among a predominantly Puerto Rican sample during the pandemic. The diversity of the sample is intentional to support an examination of Puerto Ricans and facilitate a comparison with their non-Hispanic/Latino peers within the same community. The paper aims to answer three questions: What indicators are associated with food security? What factors influence food assistance utilization? To what degree did Holyoke residents rely on aid before the pandemic and in 2022?

Holyoke is a post-industrial city in Western Massachusetts. Table 1 displays the demographic and household attributes of Holyoke, Hampden County, Massachusetts, and the United States. In 2020, the city’s population was 38,238; 51.7% of the residents were of Hispanic/Latino heritage. Holyoke has the largest per capita Puerto Rican demographic of any U.S. city. Approximately 92.7% of this city’s Hispanic/Latino population identify as Puerto Rican, comprising 45.9% of the city’s makeup [48,99].

Holyoke has a significantly higher proportion of low-income residents than Massachusetts and the United States. For example, the city’s poverty rate of 26% is more than twice the state and national rates. In 2022, the median household income in Holyoke was USD 49,007. In Massachusetts, nearly one-third of Puerto Ricans live in poverty, with an average salary of USD 31,145 [99,100,101,102].

Our data, collected in 2022, are particularly pertinent, as they coincided with a spike in inflation and food insecurity rates in Hampden County and the rest of the country. The consumer price index rose to 9.1% that year. From June 2021 to June 2022, food prices surged by 12.2%, electricity rose by 13.7%, gasoline prices jumped by 60.2%, and fuel oils went up by 70.4% [103]. Feeding America records that in 2020, 12.2% of Hampden County residents were food insecure. The rate among Hispanic/Latino residents was 24.0%. In 2021, there was a marginal improvement as the county’s rate declined to 10.9% and the Hispanic/Latino rate to 22.0%. However, Hampden County’s rate climbed to 13.3% in 2022, and the Hispanic/Latino rate soared to 28.0% [104].

Holyoke has the second-highest percentage of SNAP recipients (35.4%) in the Commonwealth of Massachusetts [102]. Many residents seek help from Feeding America’s Food Bank of Western Massachusetts [105,106,107,108]. The municipality has partnered with Nuestra Comida, a local organization, to improve food access among Hispanic/Latino communities [109]. Nuestras Raices, a non-profit focused on community and economic development, also works to improve the quality of school meals by serving locally grown food [110].

Although the food safety net is often a vital lifeline for many, these programs have barriers that stymie access [92,111,112,113,114,115,116,117,118]. This leads some families, especially SNAP recipients, to travel long distances to shop at the most affordable food stores [119,120]. In the case of Holyoke, a study found that 65.1% of SNAP recipients had access to an eligible grocery store/supermarket within a mile of their residences but frequently used their benefits at supermarkets in neighboring cities farther away from their homes [121].

Other studies highlight how the pandemic exacerbated food inaccessibility and insecurity [83,122,123,124,125,126]. In Massachusetts, surveys reveal that income, racial background, ethnic identity, education, food environment, and relationship status were related to food insecurity outcomes and the need for food aid. For example, Black and Hispanic/Latino residents and low-income adults experienced elevated food insecurity. Black and Hispanic/Latino adults also reported frequent food pantry usage at the Greater Boston Food Bank [127,128,129].

To our knowledge, this is the first paper to investigate the intricate relationships between food security, participation in assistance programs, and access needs in Holyoke, Massachusetts. The research provides a basis for understanding how a respondent’s identity and household attributes impact food insecurity, accessibility, and coping strategies. This unique study also contributes to our knowledge about federal and emergency food program usage in the Puerto Rican diaspora.

## 2. Materials and Methods

### 2.1. Survey Data Compilation

We sampled residents of Holyoke who were 18 years and over at various community locations. This study employed a variety of sampling and recruitment strategies to identify a diverse pool of eligible participants. The principal investigator engaged with community members while volunteering at food access programs. (Supplementary Materials S1 features a map illustrating the emergency food environment in Holyoke, Massachusetts, as of 2022 (see Figure S1). This section also details the methodology and the descriptive statistics summarizing the distribution of each category of outlet). We selected these sites to help us understand the community food assistance landscape and the people who use them. We recognized that although there is a need, some individuals choose not to seek food assistance. Therefore, we collected data at food distribution venues, community spaces, and events, including farmers’ markets, parks, and college campuses. To ensure a robust sample of Puerto Ricans, we used purposive sampling to identify potential respondents at community spaces, music festivals, and Puerto Rican-owned or operated restaurants.

In addition, we used a snowball sampling technique to diversify the sample by asking respondents to refer others who might be interested in the study to us. The combination of community participatory recruitment and purposive and snowball sampling generated eligible participants who were invited to complete an online Qualtrics survey using a QR code or could take the paper survey. Survey respondents could enter a raffle for USD 25 gift cards to local food retailers. A total of 381 surveys were collected, and 284 were complete to be analyzed in this paper.

### 2.2. Food Insecurity Screening

The USDA Household Food Security Six-Item Survey Module measures food security categories. The instrument contains items about the respondent’s food access. Household food security is classified according to the number of affirmative answers respondents give. The categories are (1) food security (none), (2) marginal food security (one response), (3) low food security (two to four responses), and (4) very low food security (five or more responses). This survey module is widely used in food insecurity studies [130,131].

### 2.3. Statistical Analyses

The surveys were exported, cleaned, coded, and analyzed in IBM SPSS Statistics Version 28, and tables were constructed in Microsoft Excel 16.86.2. We investigated eight independent and eleven dependent variables (see Supplementary Materials S2). We used descriptive statistics to calculate the counts, variable distributions, sample characteristics, demographics, and household attributes (Supplementary Materials S3).

In our analyses, we computed chi-square tests, binary logistic regression models, multinomial logistic regressions, and hierarchical models [132]. These predictive models quantified the unique contribution of each independent variable to the outcomes [133]. The hierarchical models estimate how groups of demographic, household, and socioeconomic variables contributed to food insecurity outcomes [134,135]. The study assessed the variance inflation factor (VIF) for multicollinearity with a threshold of 2.5 or higher.

Many studies identify race, household type, ethnicity, educational level, income, and gender identity as significant determinants of food access, engagement with nutrition programs, and food insecurity [136,137,138]. We also focused on heritage because of its significance in navigating food environments and health challenges in Hispanic/Latino communities [139,140,141]. We also explored multifamily living arrangements because the Hispanic/Latino population reports the highest multifamily co-residence rate among ethnic groups in the U.S. [142].

We ran eight binary logistic regression models. The formula is

$$ln\;(\frac{\hat{p}}{1-\hat{p}})=b_{GI}+b_{AI}+b_{EM}+b_{ED}+b_{CH}+b_{HT}+b_{HI}+b_{PR}$$

The multinomial logistic regression model formula is

$$log\;(\frac{p_{FS=j}}{p_{FS=ref}})=b_{GI}+b_{AI}+b_{EM}+b_{ED}+b_{CH}+b_{HT}+b_{HI}+b_{PR}$$

We developed one multinomial regression model for each independent variable. Owing to the limited number of high and marginal food security participants, these two categories were collapsed into high or marginal food security. The dependent variable represents households’ food security, classified into three levels: high or marginal, low, and very low. The formula details log odds of the probability of a specific food security status category (FS=j) relative to the reference category (FS=ref). The reference category consists of participants who were screened as experiencing high or marginal food security. The coefficient denotes the impact of each independent variable on the log odds of being in a specific food security category.

We employed hierarchical linear regression techniques to quantify the effect of demographic, household, and socioeconomic variables on food security status. The sequential entry of block predictors helped determine the variance explained by each set of variables. The following formulas represent each stage of the model, with variables added incrementally to evaluate their contribution to the overall predictive influence of the model:

Block 1:

$$\hat{FS}{=b}_{GI}+b_{AI}+b_{PR}+\epsilon$$

Block 2:

$$\hat{FS}={\;b}_{GI}+b_{AI}+b_{PR}+b_{CH}+b_{HT}+\epsilon$$

Block 3:

$$\hat{FS}={{\;b}_{GI}+b_{AI}+b_{PR}+b_{CH}+b_{HT}\;b}_{EM}+b_{ED}+b_{HI}+\epsilon$$

## 3. Results

### 3.1. Food Security Status

Food insecurity is a concerning issue for most of the sample; 48.6% of the respondents experienced very low food security and 24.6% low food security. Hence, 73.2% of the respondents were food insecure. The remainder (26.8%) indicated limited to no challenges in meeting their dietary intake needs.

**Figure 1:**
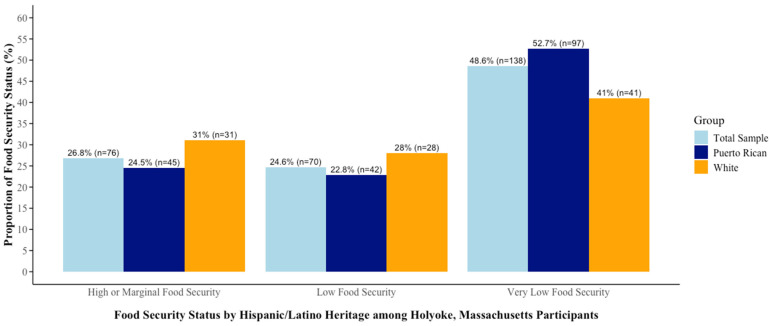
Household food security status by Hispanic/Latino heritage among participants in Holyoke, Massachusetts, 2022 (*n* = 284).

Differences in food insecurity were observed between Puerto Rican and White households. Approximately 52.7% of Puerto Ricans and 41% of White participants experienced very low food security. Additionally, 22.8% of Puerto Ricans and 28% of White people had low food security (see Figure 1). 

### 3.2. Bivariate Analyses

#### 3.2.1. Age

Age significantly impacted three dependent variables (Table 2). The youngest respondents were more prone to screening very low security (66.7%) than those 30–39 years old (41.2%) and 40 years or older (39.1%). These differences were statistically relevant (χ^2^ = 17.751, *df* = 4, *p* < 0.001). We found that COVID-19 significantly altered the federal food assistance needs of the respondents. Age was significant for determining the use of government aid (χ^2^ = 20.318, *df* = 2, *p* < 0.001). Age also emerged as a critical predictor of eating prepared meals at senior centers or programs. Survey participants who were 40 years or older (48.9%) and those who were 30–39 years old (47.1%) accessed senior centers or programs for food at lower rates than the youngest respondents (71.1%). These variations were statistically significant (χ^2^ = 13.422, *df* = 2, *p* < 0.001).

#### 3.2.2. Income

Income had significant bearings on six dependent variables (Table 3). Therefore, respondents with incomes of USD 0–USD 49,999 were likelier to experience very low food security (59.0%) than those with higher incomes (32.4%). These outcomes were statistically significant (χ^2^ = 20.854, *df* = 2, *p* < 0.001). The need for federal aid during the pandemic was significantly higher among lower-income respondents (82.7%) than higher-income individuals (68.5%) (χ^2^ = 7.715, *df* = 1, *p* = 0.005). Additionally, 88.6% of low-income study participants experienced disruptions in emergency relief needs, while 78.1% of higher-income respondents experienced changes in emergency food access needs (χ^2^ = 5.372, *df* = 1, *p* = 0.020).

In 2022, a high correlation existed between income and current usage of assistance programs. Relying on federal food assistance programs was ubiquitous among low-income participants (74.4%) compared to those with household incomes of USD 50,000 or more (57.3%). The differences in federal food assistance usage were significant (χ^2^ = 9.024, *df* = 1, *p* = 0.003). The results indicate that lower-income households (60.7%) are more inclined to seek assistance at senior facilities than households with an annual income of USD 50,000 or more (46.8%). The chi-square results suggest these variations were statistically valid (χ^2^ = 5.244, *df* = 1, *p* = 0.022). Similarly, using these outlets for emergency food was significantly higher among lower-income respondents (71.7%) than among higher-income study participants (54.1%) (χ^2^ = 9.204, *df* = 1, *p* = 0.002).

#### 3.2.3. Hispanic/Latino Heritage

The sample contains 184 Puerto Ricans (64.8%) and 100 non-Hispanic/Latino White respondents (35.2%). Hispanic/Latino ancestry was strongly associated with using food assistance programs (Table 4). Puerto Ricans (76.1%) relied more heavily on federal support than White participants (57%) (χ^2^ = 11.108, *df* = 1, *p* < 0.001). Additionally, the pandemic altered federal food assistance needs more for Puerto Ricans (81.0%) than White respondents (70.0%) (χ^2^ = 4.424, *df* = 1, *p* = 0.035). Concomitantly, Puerto Rican respondents (71.4%) reported higher use of government support than Whites (61.0%). More than half of Puerto Rican respondents (59.2%) requested relief at senior centers, compared to White participants (48.0%).

#### 3.2.4. Gender

No significant associations were detected between gender and the elements of food access (Table 5). Despite the absence of statistical significance, some descriptive findings are noteworthy. Most women participants reported very low food security (52.1%) compared to men (45.3%). Men (71.5%) were likelier to rely on federal relief than women (67.1%). However, during the pandemic, government aid was higher among women (71.2%) than men (63.7%).

#### 3.2.5. Employment

Employment was a robust predictor of food security outcomes and federal food assistance usage. Most unemployed participants (67.6%) and over half of part-time respondents (54.3%) had very low food security (χ^2^ = 20.294, *df* = 4, *p* < 0.001). A significant correlation exists between employment and federal assistance (χ^2^ = 8.046, *df* = 2, *p*= 0.018). Higher percentages of part-time (89.1%) and unemployed individuals (82.4%) reported that the pandemic modified their need for federal assistance than those employed full-time (71.3%). Employment status was also a strong predictor of government aid (χ^2^ = 6.059, *df* = 2, *p* = 0.048), where unemployed (71.6%), part-time (80.4%), and full-time participants (62.3%) relied on government assistance for food access needs (Table 6).

#### 3.2.6. Educational Attainment

Education had profound impacts on nine dependent variables (Table 7). The results indicate a robust association between education and food security (χ^2^ = 29.997, *df* = 4, *p* < 0.001). Individuals with a university degree were less likely to experience very low food security (27.8%) than those with a technical diploma (48.1%) or a secondary school certificate or less (71.1%).

Lower educational attainment was linked to higher use of federal aid (χ^2^ = 15.643, *df* = 2, *p* < 0.001). The data reveal a significant contrast; individuals with a secondary school diploma or less were likelier to use federal support (86.8%) than those with a university degree (59.5%). The results also show a strong association between education and the effects of the pandemic on federal assistance needs (χ^2^ = 10.998, *df* = 2, *p* = 0.004). Individuals with secondary education or less were more prone to document that the pandemic altered their need for government assistance (90.8%) than those who completed college (72.2%). The bivariate analysis also shows a robust association between education and federal relief (χ^2^ = 12.442, *df* = 2, *p* = 0.002). The data show that individuals with a secondary diploma or less were more inclined to use federal support (84.0%) than those with associate, technical, or some college education (62.5%) or who completed college (60.8%).

Educational attainment was significantly tied to emergency food assistance needs (χ^2^ = 13.161, *df* = 2, *p* < 0.001). The pandemic affected the food access needs of virtually all individuals with only a secondary school education or less (97.3%). The chi-square test revealed a significant association between education levels and engagement with food delivery services (χ^2^ = 7.109, *df* = 2, *p* = 0.029) and contact with senior centers (χ^2^ = 24.653, *df* = 2, *p* < 0.001). Similarly, education was a significant factor in obtaining food from food pantries and banks (χ^2^ = 13.820, *df* = 2, *p* < 0.001) and soup kitchen or shelter visits (χ^2^ = 13.624, *df* = 2, *p* < 0.001).

#### 3.2.7. Children in the Household

Children were crucial in determining food security status and reliance on community aid. Living with children was correlated with food security (χ^2^ = 7.382, *df* = 2, *p* = 0.025). Households without dependents tended to have high or marginal food security (40.0%), while only 23.2% of the families with children had this classification. The pandemic’s impacts on emergency food access depended on whether there were dependents in the home (χ^2^ = 19.498, *df* = 1, *p* < 0.001). COVID-19 significantly impacted the emergency food access needs of homes with children (89.6%) compared to living arrangements without (66.1%). Having children in the household was also strongly connected to emergency meal deliveries (χ^2^ = 6.556, *df* = 1, *p* = 0.010) and community kitchen use (χ^2^ = 13.058, *df* = 1, *p* < 0.001) (Table 8).

#### 3.2.8. Household Type

Table 9 shows that household type significantly correlated with food security status and emergency food access. The bivariate analysis revealed robust ties between household type and food security categories (χ^2^ = 17.380, *df* = 8, *p* = 0.026). Approximately 60% of multifamily and single-parent households undergo very low food security. The household type was also related to awareness of community-based emergency food outlets (χ^2^ = 14.606, *df* = 4, *p* = 0.006). Single-parent households were the most knowledgeable about local emergency assistance (91.4%). The results indicate household type is significant for visiting senior centers and programs (χ^2^ = 10.265, *df* = 4, *p* = 0.036).

### 3.3. Binary Logistic Regression Models

#### 3.3.1. Federal Food Assistance Usage Before COVID-19

The binary logistic regression results signify that for respondents 40 years and over, the odds of pre-pandemic federal assistance usage were 51.0% lower than for 18–29-year-olds (OR = 0.490; CI = 0.246–0.975, *p* = 0.042). The model predicts White participants’ odds of using federal assistance are 49.8% lower (OR = 0.502; CI = 0.285–0.886, *p* = 0.018 *) than Puerto Ricans. The results also suggest that for those with some university education, the chances of enrolling in federal relief are 75.2% lower (OR = 0.248; CI = 0.102–0.605, *p* = 0.002) than those with secondary schooling or less (Table 10).

#### 3.3.2. COVID-19 Altered Federal Food Assistance Needs

Table 11 illustrates that for respondents aged 30–39, the odds of COVID-19 altering federal food assistance needs were 83.3% lower (OR = 0.167; CI = 0.067–0.416, *p* ≤ 0.001) compared to those aged 18–29. The results also signify that for older respondents (40 years or older), the risk of COVID-19 altering federal food assistance needs decreased by 71.4% (OR = 0.286; CI = 0.133–0.615, *p* ≤ 0.001) more than for those aged 18 to 29. The model predicts that for households with incomes of USD 50,000 or more, the odds of the pandemic altering federal food assistance needs were 52.6% lower (OR = 0.474; CI = 0.233–0.963, *p* = 0.039) than for those with salaries between USD 0 and 49,999. The findings state that cohabitating with a partner meant the probability of COVID-19 altering federal food assistance needs was 78.0% lower than for those living in a single-parent domicile (OR = 0.220; CI = 0.055–0.882, *p* = 0.033).

#### 3.3.3. How COVID-19 Altered Emergency Food Assistance Needs

Table 12’s results signify that for respondents with some college education, the odds of COVID-19 altering emergency food assistance needs are 82.3% lower than those who did not attend university (OR = 0.177; CI = 0.036–0.880, *p* = 0.034). The results show that the chances of COVID-19 altering the emergency food assistance needs for households with no children are 73.9% less probable than for homes with children (OR = 0.261; CI = 0.109–0.625, *p* = 0.003).

#### 3.3.4. COVID-19 Altered Food Access

Table 13 reveals that for respondents aged 40 or older, the odds of COVID-19 altering food access had a reduced chance of 73.1% compared to participants aged 18 to 29 (OR = 0.269; CI = 0.098–0.742, *p* = 0.011).

#### 3.3.5. Federal Food Aid in 2022

Table 14 demonstrates that the odds of using federal support for participants with no children in their household are 55.6% lower than for participants living with no children (OR = 0.444; CI = 0.209–0.943, *p* = 0.035).

#### 3.3.6. Awareness of Emergency Food Outlets in a Community

The findings show that participants with some university education had a 65.6% decreased likelihood of knowing about community-based emergency food assistance compared with those who did not attend university (OR = 0.344; CI = 0.131–0.905, *p* = 0.031). Respondents living in two-parent households are 81.4% less inclined to know about community emergency food resources than individuals residing in single-guardian arrangements (OR = 0.186; CI = 0.043–0.806, *p* = 0.025). The model also suggests that the probability of knowledge about local emergency aid resources was 75.0% lower for respondents living in multifamily households than those in single-parent situations (OR = 0.250; CI = 0.079–0.787, *p* = 0.018). Households with roommates had a 73.3% decreased likelihood of knowing about emergency resources in their community compared with respondents in single-parent domiciles (OR = 0.267; CI = 0.090–0.794, *p* = 0.018) (Table 15).

#### 3.3.7. Utilization of Emergency Meal Delivery Provisions

Table 16 suggests that participants who completed their college degrees were 46.8% less likely to use meal delivery services than those who did not attend university (OR = 0.532; CI = 0.287–0.989, *p* = 0.046). The model reveals that the chances of opting for delivered meals were 51.5% lower in households with no children than for respondents living in homes with children (OR = 0.485; CI = 0.238–0.988, *p* = 0.046).

#### 3.3.8. Seeking Prepared Meals from Programs and Senior Centers

Table 17 displays that for respondents aged 30–39, the likelihood of seeking relief at senior centers is reduced by 51.4% compared with participants aged 18–29 years (OR = 0.486; CI = 0.253–0.933, *p* = 0.030). The findings indicate that for those who finished some college and those with an associate degree or technical certificate, the probability of eating prepared meals at senior facilities is 69.0% lower than for participants with a secondary school diploma or less (OR = 0.310; CI = 0.1456–0.664, *p* = 0.003). The findings demonstrate that for participants with a college background or more, the chances of requesting aid at senior facilities are 62.4% lower than for participants with a high school diploma or less (OR = 0.376; CI = 0.201–0.702, *p* = 0.002).

#### 3.3.9. Obtaining Assistance from Pantries or Banks

The regression model indicates that for individuals who attended but did not complete university, the chances of visiting pantries or banks are 78.3% lower than for respondents with the lowest educational attainment (OR = 0.217; CI = 0.076–0.617, *p* = 0.004). The model signifies that the probability of requesting support from pantries or banks for households without children is 60.7% lower than for homes with children (OR = 0.393; CI = 0.173–0.890, *p* = 0.025). The data also uncovered that two-parent households are 4.999 times more likely to seek support from pantries or banks than single-parent ones (OR = 4.999; CI = 1.223–20.422, *p* = 0.025) (Table 18).

#### 3.3.10. Reliance on a Soup Kitchen or Shelter

Table 19 denotes that for respondents in domiciles with annual incomes of USD 50,000 or more, the odds of using these emergency services were 54.3% lower than for those in domiciles with annual incomes of less than USD 50,000 (OR = 0.457; CI = 0.251–0.831, *p* = 0.010). Respondents who attended college but did not complete it were 60.2% more likely than those who never attended university to seek kitchen services (OR = 0.398; CI = 0.179–0.886, *p* = 0.024). Households with no children had a 57.7% reduced likelihood compared with individuals living with children (OR = 0.423; CI = 0.206–0.867, *p*= 0.019).

### 3.4. Multivariate Analyses of Food Security Status

#### 3.4.1. Multinomial Regression Analysis

This model analyzes the effect of demographic and household features on the low (has difficulty meeting nutritional needs but not going hungry) or very low (inability to satisfy dietary needs and going hungry) food security categories, denoting food insecurity incidents.

Educational attainment and household composition were significant indicators of low food security status. The results show that for respondents who did not attend university, the relative risk ratio (RRR) of being classified as having low food security decreased by 0.323 in comparison to participants who graduated from university (RRR = 0.323; CI = 0.105–0.994, *p* = 0.049). Conversely, a high proportion of people in living arrangements with children experienced low food security. The relative risk ratio for experiencing low food security increased fourfold for those residing in domiciles with children (RRR = 4.010; CI = 1.398–11.500, *p* = 0.009).

Age, income, and employment status were strong predictors of undergoing food insecurity with hunger. The findings denote that the relative risk ratio of being classified as very low rose by 2.989 for respondents aged 18–29 (RRR = 2.989; CI = 1.299–6.877, *p* = 0.009). Similarly, for respondents aged 30 to 39, the relative risk ratio increased by a factor of 2.856 (RRR = 2.856; CI = 1.248–6.535, *p* = 0.012). Similarly, for participants whose household income is below USD 50,000, the relative risk ratio increased by 2.768 (RRR = 2.768, CI = 1.346–5.689, *p* = 0.006). Additionally, the relative risk ratio dropped by 0.235 for respondents who work full-time (RRR = 0.235; CI = 0.095- 0.584, *p ≤* 0.001) (Table 20).

#### 3.4.2. Hierarchical Linear Regression Models

We constructed hierarchical linear regressions, inserting new blocks of variables into the models to measure how demographic, household, and socioeconomic descriptors predict and influence food security status. These variables were entered into three blocks to evaluate the incremental changes in variance in each model.

Model 1 contains demographic variables—age, gender, and Hispanic/Latino heritage. It explained 5.4% of the variance in food security (R^2^ = 0.054, *p* = 0.001). Age was the only significant independent variable in the model (β = 0.208, *p* = 0.001). Second, we introduced household variables—household type and children in the household—into Model 2. The additions increased the variance to 7.0% (R^2^ = 0.070, ΔR^2^ = 0.016, *p* = 0.095). The new variables were insignificant in the model. However, age remained significant, negatively impacting food security outcomes. In the final step, we added socioeconomic variables—income, educational background, and employment status—to Model 3. This resulted in the model accounting for 19.1% of the variance (R^2^ = 0.191, ΔR^2^ = 0.121, *p* < 0.001). Income (β = –0.301, *p*= 0.003), education (β = −0.164, *p*= 0.014), age (β = −0.171, *p* = 0.004), and employment (β = –0.188, *p* = 0.001) had negative impacts on food security status (Table 21).

## 4. Discussion

This paper analyzed how demographic and household characteristics were related to federal and emergency aid, food insecurity status, and food access needs in Holyoke, Massachusetts, amidst the pandemic. The timing of the research is crucial, as 2022 marked the historical rise in national food insecurity rates [21]. The sample was predominantly Puerto Rican (64.8%). We focused on this sub-sample because prior research indicated that this group faces chronic poverty, high food insecurity, and pervasive health concerns [143,144]. Hence, it is imperative that we fully understand the vulnerabilities of Puerto Ricans, access challenges, and coping strategies.

This study lends credence to earlier claims that the pandemic profoundly altered the food system and created new challenges to achieving food security domestically and globally [145]. Our results indicate that most of the sample encountered interferences that modified their need for federal (77.1%) and emergency (84.5%) food assistance. These findings underscore the multi-scalar impacts of the pandemic and how it severely affected food access in Holyoke. Over two-thirds of the sample (73.2%) experienced food insecurity and reported that the pandemic made it challenging to meet their nutritional needs consistently. Consequently, respondents relied on government and community assistance programs more heavily than usual [146]. The heightened demand for food assistance persisted into 2022. Our findings not only help to elucidate the effects of COVID-19 on Holyoke residents’ food access, but this study also highlights inequities in the distribution of impacts. In short, our study supports the findings of other researchers who show that demographic and household factors converged to influence food insecurity outcomes, food assistance participation, and food access needs [147,148].

### 4.1. Household Factors [143,144]

Our findings demonstrate that household attributes are robust predictors of food access [28,149,150]. There was an inverse relationship between households without children and those needing and using food assistance programs. Households without children had a lower inclination to use emergency food relief than other households, while households with children were more susceptible to using food assistance programs. These findings run counter to those of other scholars reporting a decline in food assistance participation in homes with children during the COVID-19 period [28,151,152].

Respondents living in households with children were at heightened risk for food insecurity. We found that such participants were four times more likely to be classified as having low food security. The USDA also observed the vulnerability of households with children and a pandemic rise in food insecurity from 2021 to 2022—from 6.2% (2.3 million) to 8.8% (3.3 million) [21]. Thus, household composition significantly affects the occurrences of food insecurity.

The USDA consistently reports that single-parent households exhibit increased occurrences of food insecurity [21]. Our study confirms that the household structure is critical in food assistance participation. The analysis points to the vulnerabilities of different household types. It reveals that two-parent households had a significant positive association with seeking aid from pantries or other food repositories. This finding suggests that even two-parent households, often perceived as more stable than single-parent households, are nutritionally vulnerable and fall back on food assistance. The results also uncovered a relationship not identified in earlier studies; the household type was associated with the likelihood of being aware of community emergency food networks. Those living in multifamily households, alone or with roommates, and in two-parent households had lower awareness of such institutions and programs. These findings imply that communication barriers or constrained outreach efforts may hinder the ability of some households to find out about the resources.

The results indicate a strong correlation between income and food access. Participants with household incomes of USD 50,000 or more showed significant negative associations with needing federal support and visiting soup kitchens or shelters. The highest-income households were least likely to require assistance, as their income protected them from food insecurity. In contrast, households with incomes under USD 50,000 had a high likelihood of facing food insecurity; they were about 2.8 times more likely than high-income households. These findings support the claims that higher income levels are associated with greater access to nutritious food [96,153,154,155].

### 4.2. Demographic Factors

Although gender identity was not statistically significant, notable differences were evident in the descriptive findings. For example, women showed elevated levels of food insecurity compared with men. We surmise that the gendered nature of food insecurity relates to structural factors not explored in this study. Women frequently serve as primary or sole caregivers for children and other dependents. As household caregivers, women are often compelled to compromise their nutritional needs to prevent their children and partners from going hungry. Moreover, women are paid less and are more vulnerable to unemployment, income erosion, and dependence on social safety net programs. These vulnerabilities compound the risks of facing food insecurity. Many researchers also find that women are susceptible to high rates of food insecurity [9,156,157].

Our study found significant relationships between employment status, food security classification, and federal relief usage before and during the pandemic. The pandemic-induced economic turmoil resulted in skyrocketing unemployment that impacted Holyoke residents [48]. The hierarchical model points to the influential role of employment status in determining food insecurity. This study’s findings may reflect changes in employment that heighten food insecurity and generate new need for government assistance [158,159,160,161].

This study draws attention to the vulnerability of respondents with low educational attainment. Participants with advanced degrees were less likely to utilize various emergency food outlets and government aid before the COVID-19 crisis. Hence, we found the likelihood of resorting to emergency food assistance programs decreased as the educational level rose.

Federal aid utilization was markedly higher among Puerto Ricans than among non-Hispanic/Latino White respondents. Specifically, the logistic regressions suggest that Hispanic/Latino heritage significantly predicted federal food assistance usage. Although Puerto Rican ancestry was a significant determinant in federal food assistance usage, Hispanic/Latino heritage had a weak association with accessing emergency provisions.

Several factors may explain this conundrum. Emergency food outlets operate through different modes of grassroots outreach and include various eligibility requirements. These community programs also include varying days and hours of operation, making some people perceive these programs as a last resort and unaware of their availability. In contrast, federal food assistance provides prolonged support to eligible recipients. Typically, this relief also includes flexibility in redeeming vouchers at preferred food retailers and options for purchasing culturally desired nutritional items. Puerto Ricans’ status as U.S. citizens also may facilitate greater access to government-administered programs. Thus, the stability and widespread awareness of federal food assistance programs may explain the higher participation rates in government food programs over local emergency food relief among Puerto Rican households. This distinction is critical in helping us understand how food assistance programs differ and how community members seek and obtain relief.

This study advances our understanding of age’s enormous influence on food insecurity outcomes and food assistance enrollment. The data suggest that federal food assistance and emergency relief participation decreases as age increases. For example, we found that individuals over 30 years have a reduced probability of needing government resources due to pandemic disruptions. This age group was also less prone to obtaining relief at senior centers or experiencing an overall change in food access due to the pandemic. Though respondents aged 30–39 were less likely to rely on food aid, they still experienced very low food security rates.

Age was also a robust indicator of food security in the hierarchical models. These findings concur with other scholars who contend that age group is a crucial determinant of food security, access, and reliance on support [162,163]. The significance of demographics and household factors in determining access to assistance programs aligns with findings from other regions, such as Hialeah, Florida, a primarily Hispanic/Latino community [164].

### 4.3. Policy Implications

Disparities in food assistance participation, food accessibility, and food insecurity outcomes underscore the problem’s urgency and the pressing need for action [165,166,167]. Addressing the inequities in emergency food access requires an immediate influx of government and philanthropic dollars, individual donations, and enhanced infrastructure to bolster the capacity of emergency food organizations to meet community needs. New strategic priorities should include grassroots campaigns targeting vulnerable populations such as those identified in this study, forging partnerships with local community leaders to spread awareness, and providing culturally desirable foods to meet the diverse needs of community members [168,169,170].

Dedicated community outreach initiatives must be established in predominantly Puerto Rican and Hispanic/Latino neighborhoods to improve emergency food access. Similar outreach efforts should be conducted in other culturally distinct enclaves and low-income neighborhoods. The research shows a strong link between employment and food access; therefore, emergency food outlets should consider expanding their services to include employment services, training programs, job and educational opportunities, housing information, and nutrition education and resources.

Households with children were associated with limited access to food. To ameliorate conditions, federal, state, and local governments should enhance existing programs such as the Breakfast and School Lunch Program while expanding opportunities for children and youth to access nutritious food when school is not in session [171,172,173]. These efforts can include expanding after-school meal programs, weekend backpack food relief, and food assistance during holidays, closures, and summer breaks. The federal government should also consider reinstating popular pandemic-era programs like the Farmers to Families Food Box. These comprehensive recommendations will enhance equitable and just food assistance access while reducing food insecurity.

### 4.4. Strengths and Limitations

This study includes several strengths that contribute to the robustness of our findings. The researchers included community members in the data collection phase, ensuring that the diversity of the Holyoke community is represented and valued. Previous food access research demonstrates that food-insecure individuals do not necessarily seek food assistance for many reasons [174]. This led us to collect data from many locations to obtain a diverse sample from various cultural, social, and economic backgrounds. The survey offered three language options. Participants were also offered two options to complete the survey: online or printed copies. This made the study accessible to households with limited digital access or internet skills. Participants responded positively to these options, as some respondents preferred to write their answers on paper. Thus, this study illuminates the food access and assistance experiences of Holyoke residents while incorporating their diverse perspectives and experiences.

Notwithstanding, this study has some limitations. The survey methods, while thorough, may have excluded critical perspectives in the Holyoke area. There is a possibility that selection bias emerged when identifying eligible participants. Hence, the sample could be biased toward those eager to engage. The optional participation in the survey may result in self-selection bias, potentially missing some perspectives of Holyoke residents. While multiple languages were used to increase inclusivity, there may still be linguistic or cultural nuances that the survey design did not fully capture; this could affect the clarity and accuracy of responses. We opted to use the USDA’s six-item screening module to reduce respondent burden and bias, as it is less time-intensive while maintaining validity. However, we recognize that the 18-item module has the potential to provide more detailed household information that could enhance the comparative aspects of this study. These limitations point to the necessity of further research to understand more nuanced dimensions of food insecurity and access.

## 5. Conclusions

While the need for food assistance programs predated the COVID-19 pandemic, our study demonstrates that the pandemic worsened food insecurity and heightened the demand for government and community food assistance. Our study provides a snapshot of the food access dynamics in one small Massachusetts city in 2022; it showed that food insecurity was still pervasive two years after the pandemic began. Despite the immense infusion of government funds into food assistance, food insecurity proved deeply entrenched and difficult to alleviate. The demand for food assistance relief was high among Puerto Rican households before the pandemic and remained in 2022. Many studies report that Puerto Ricans are susceptible to food insecurity. Our analysis also identifies alarming rates of food insecurity within this community and examines various contributing factors.

The reliance on emergency food assistance programs was significantly determined by income, children living in the household, household type, educational attainment, and age. Similarly, pre-pandemic and pandemic use of federal assistance was associated with children in the household, age, and academic background. However, we identified a key difference: Puerto Rican heritage was a significant determinant of participation in federal food assistance but not emergency programs.

The implications of these findings extend beyond Holyoke, Massachusetts. It is clear from the case study that a confluence of economic, social, and cultural variables influences access to federal and emergency food aid. The demographic and household disparities determining access to assistance programs present significant food equity and justice concerns. Our study underscores the importance of expanding research endeavors to include a broader range of variables explored to understand the factors influencing food access fully. Our study sheds light on structural barriers influencing food insecurity and inaccessibility. By addressing these barriers, we can work toward a more equitable food assistance system that supports historically marginalized populations. Further investigation should explore the roles of sexual orientation, diet-related diseases, and the history of pre-existing health conditions in food access and security. Such inquiries ensure a fair, just, and equitable distribution of food assistance resources locally and nationally.

## Figures and Tables

**Table 1:** Census Demographics of the United States, Massachusetts, Hampden County, and Holyoke City.

	United States		Massachusetts		Hampden County			Holyoke City
Characteristics	Number	Percent	Number	Percent	Number	Percent	Number	Percent
Population Size	333,287,557	100.0	7,001,399	100.0	460,291	100.0	38,238	100.0
Demographic Characteristics								
Female persons	168,189,509	50.4	3,570,713	51.0	236,129	51.3	19,425	50.8
Hispanic or Latino	63,675,446	19.1	917,183	13.1	121,319	27.7	19,769	51.7
Socioeconomic Status								
High school graduate or higher (persons age 25 years+)	296,148,349	88.9	6,385,276	91.2	399,072	86.7	30,132	78.8
Bachelor’s degree or higher (persons age 25 years+)	112,548,632	33.7	3,213,642	45.9	131,643	28.6	8489	22.2
Persons in poverty	38,399,069	11.5	728,145	10.4	77,789	16.9	9942	26.0
Household Arrangement								
Households	124,010,992	37.2	2,740,995	100.0	183,380	100.0	15,112	100.0
Language other than English spoken at home (persons age 5 years+)	72,370,775	21.7	671,544	24.5	48,596	26.5	6453	42.7

Compiled from [48,99].

**Table 2:** The Relationship Between Age, Food Security Outcomes, and Using Emergency and Federal Food Assistance During COVID-19 in Holyoke, Massachusetts.

Experiences of Food Security, Emergency Food Services, and Federal Food Assistance	Total Sample		18–29 Years Old		30–39 Years Old		40 Years or Older		χ^2^	*df*	Significance (*p*-Value)
	Number	Percent	Number	Percent	Number	Percent	Number	Percent			
USDA food security category	284	100.0	90	100.0	102	100.0	92	100.0	17.751	4	<0.001 ***
High or Marginal Food Security	76	26.8	15	16.7	30	29.4	31	33.7			
Low Food Security	70	24.6	15	16.7	30	29.4	25	27.2			
Very Low Food Security	138	48.6	60	66.7	42	41.2	36	39.1			
Used federal food assistance programs prior to COVID-19	284	100.0	90	100.0	102	100.0	92	100.0	4.981	2	0.083
Yes	197	69.4	68	75.6	73	71.6	56	60.9			
No	87	30.6	22	24.4	29	28.4	36	39.1			
COVID-19 altered federal food assistance need	284	100.0	90	100.0	102	100.0	92	100.0	20.318	2	<0.001 ***
Yes	219	77.1	82	91.1	79	77.5	58	63.0			
No	65	22.9	8	8.9	23	22.5	34	37.0			
COVID-19 altered emergency food assistance need	271	100.0	88	100.0	99	100.0	84	100.0	4.132	2	0.127
Yes	229	84.5	79	89.8	84	84.8	66	78.6			
No	42	15.5	9	10.2	15	15.2	18	21.4			
COVID-19 altered food access	273	100.0	89	100.0	99	100.0	85	100.0	5.744	2	0.057
Yes	238	87.2	81	91.0	89	89.9	68	80.0			
No	35	12.8	8	9.0	10	10.1	17	20.0			
Use federal food assistance programs in the last 30 days	282	100.0	90	100.0	100	100.0	92	100.0	3.704	2	0.157
Yes	191	67.7	68	75.6	64	64.0	59	64.1			
No	91	32.3	22	24.4	36	36.0	33	35.9			
Aware of emergency food program in their community	284	100.0	90	100.0	102	100.0	92	100.0	1.512	2	0.470
Yes	226	79.6	69	76.7	80	78.4	77	83.7			
No	58	20.4	21	23.3	22	21.6	15	16.3			
Use emergency food assistance for meal delivery services in the last 30 days	284	100.0	90	100.0	102	100.0	92	100.0	3.201	2	0.202
Yes	182	64.1	64	71.1	60	58.8	58	63.0			
No	102	35.9	26	28.9	42	41.2	34	37.0			
Visit programs and senior centers for prepared meals in the last 30 days	284	100.0	90	100.0	102	100.0	92	100.0	13.422	2	<0.001 ***
Yes	157	55.3	64	71.1	48	47.1	45	48.9			
No	127	44.7	26	28.9	54	52.9	47	51.1			
Obtain food from a church pantry, food pantry, or food bank in the last 30 days	284	100.0	90	100.0	102	100.0	92	100.0	4.692	2	0.096
Yes	228	80.3	79	87.8	78	76.5	71	77.2			
No	56	19.7	11	12.2	24	23.5	21	22.8			
Visit a soup kitchen or shelter for emergency food in the last 30 days	284	100.0	90	100.0	102	100.0	92	100.0	4.217	2	0.121
Yes	184	64.8	66	73.3	62	60.8	56	60.9			
No	100	35.2	24	26.7	40	39.2	36	39.1			

Significance levels (*p*-values): *** α ≤ 0.001.

**Table 3:** The Relationship Between Income, Food Security Outcomes, and Using Emergency and Federal Food Assistance During COVID-19 in Holyoke, Massachusetts.

Experiences of Food Security, Emergency Food Services, and Federal Food Assistance	Total Sample		USD 0–49,999		USD 50,000 or More		χ^2^	*df*	Significance (*p*-Value)
	Number	Percent	Number	Percent	Number	Percent			
USDA food security category	284	100.0	173	100.0	111	100.0	20.854	2	<0.001 ***
High or Marginal Food Security	76	26.8	33	19.1	43	38.7			
Low Food Security	70	24.6	38	22.0	32	28.8			
Very Low Food Security	138	48.6	102	59.0	36	32.4			
Used federal food assistance programs prior to COVID-19	284	100.0	173	100.0	111	100.0	2.503	1	0.114
Yes	197	69.4	126	72.8	71	64.0			
No	87	30.6	47	27.2	40	36.0			
COVID altered federal food assistance need	284	100.0	173	100.0	111	100.0	7.715	1	0.005 **
Yes	219	77.1	143	82.7	76	68.5			
No	65	22.9	30	17.3	35	31.5			
COVID altered emergency food assistance need	271	100.0	166	100.0	105	100.0	5.372	1	0.020 *
Yes	229	84.5	147	88.6	82	78.1			
No	42	15.5	19	11.4	23	21.9			
COVID altered food access	273	100.0	167	100.0	106	100.0	1.605	1	0.205
Yes	238	87.2	149	89.2	89	84.0			
No	35	12.8	18	10.8	17	16.0			
Use federal food assistance programs in the last 30 days	282	100.0	172	100.0	110	100.0	9.024	1	0.003 **
Yes	191	67.7	128	74.4	63	57.3			
No	91	32.3	44	25.6	47	42.7			
Aware of emergency food program in their community	284	100.0	173	100.0	111	100.0	2.586	1	0.108
Yes	226	79.6	143	82.7	83	74.8			
No	58	20.4	30	17.3	28	25.2			
Use emergency food assistance for meal delivery services in the last 30 days	284	100.0	173	100.0	111	100.0	0.048	1	0.826
Yes	182	64.1	110	63.6	72	64.9			
No	102	35.9	63	36.4	39	35.1			
Visit programs and senior centers for prepared meals in the last 30 days	284	100.0	173	100.0	111	100.0	5.244	1	0.022 **
Yes	157	55.3	105	60.7	52	46.8			
No	127	44.7	68	39.3	59	53.2			
Obtain food from a church pantry, food pantry, or food bank in the last 30 days	284	100.0	173	100.0	111	100.0	0.905	1	0.341
Yes	228	80.3	142	82.1	86	77.5			
No	56	19.7	31	17.9	25	22.5			
Visit a soup kitchen or shelter for emergency food in the last 30 days	284	100.0	173	100.0	111	100.0	9.204	1	0.002 **
Yes	184	64.8	124	71.7	60	54.1			
No	100	35.2	49	28.3	51	45.9			

Significance levels (*p*-values): * α ≤ 0.05, ** α ≤ 0.01, *** α ≤ 0.001.

**Table 4:** The Relationship Between Puerto Rican Identity, Food Security Outcomes, and Using Emergency and Federal Food Assistance During COVID-19 in Holyoke, Massachusetts.

Experiences of Food Security, Emergency Food Services, and Federal Food Assistance	Total Sample		Puerto Rican		White		χ^2^	*df*	Significance (*p*-Value)
	Number	Percent	Number	Percent	Number	Percent			
USDA food security category	284	100.0	184	100.0	100	100.0	3.571	2	0.168
High or Marginal Food Security	76	26.8	45	24.5	31	31.0			
Low Food Security	70	24.6	42	22.8	28	28.0			
Very Low Food Security	138	48.6	97	52.7	41	41.0			
Used federal food assistance programs prior to COVID-19	284	100.0	184	100.0	100	100.0	11.108	1	<0.001 ***
Yes	197	69.4	140	76.1	57	57.0			
No	87	30.6	44	23.9	43	43.0			
COVID altered federal food assistance need	284	100.0	184	100.0	100	100.0	4.424	1	0.035 *
Yes	219	77.1	149	81.0	70	70.0			
No	65	22.9	35	19.0	30	30.0			
COVID altered emergency food assistance need	271	100.0	178	100.0	93	100.0	2.630	1	0.105
Yes	229	84.5	155	87.1	74	79.6			
No	42	15.5	23	12.9	19	20.4			
COVID altered food access	273	100.0	180	100.0	93	100.0	0.169	1	0.681
Yes	238	87.2	158	87.8	80	86.0			
No	35	12.8	22	12.2	13	14.0			
Use federal food assistance programs in the last 30 days	282	100.0	182	100.0	100	100.0	3.211	1	0.073
Yes	191	67.7	130	71.4	61	61.0			
No	91	32.3	52	28.6	39	39.0			
Aware of emergency food program in their community	284	100.0	184	100.0	100	100.0	1.858	1	0.173
Yes	226	79.6	142	77.2	84	84.0			
No	58	20.4	42	22.8	16	16.0			
Use emergency food assistance for meal delivery services in the last 30 days	284	100.0	184	100.0	100	100.0	0.000	1	0.983
Yes	182	64.1	118	64.1	64	64.0			
No	102	35.9	66	35.9	36	36.0			
Visit programs and senior centers for prepared meals in the last 30 days	284	100.0	184	100.0	100	100.0	3.311	1	0.069
Yes	157	55.3	109	59.2	48	48.0			
No	127	44.7	75	40.8	52	52.0			
Obtain food from a church pantry, food pantry, or food bank in the last 30 days	284	100.0	184	100.0	100	100.0	1.787	1	0.181
Yes	228	80.3	152	82.6	76	76.0			
No	56	19.7	32	17.4	24	24.0			
Visit a soup kitchen or shelter for emergency food in the last 30 days	284	100.0	184	100.0	100	100.0	0.971	1	0.324
Yes	184	64.8	123	66.8	61	61.0			
No	100	35.2	61	33.2	39	39.0			

Significance levels (*p*-values): * α ≤ 0.05, *** α ≤ 0.001.

**Table 5:** The Relationship Between Gender, Food Security Outcomes, and Using Emergency and Federal Food Assistance During COVID-19 in Holyoke, Massachusetts.

Experiences of Food Security, Emergency Food Services, and Federal Food Assistance	Total Sample		Women		Men		χ^2^	*df*	Significance (*p*-Value)
	Number	Percent	Number	Percent	Number	Percent			
USDA food security category	283	100.0	146	100.0	137	100.0	1.377	2	0.502
High or Marginal Food Security	75	26.5	37	25.3	38	27.7			
Low Food Security	70	24.7	33	22.6	37	27.0			
Very Low Food Security	138	48.8	76	52.1	62	45.3			
Used federal food assistance programs prior to COVID-19	283	100.0	146	100.0	137	100.0	0.645	1	0.422
Yes	196	69.3	98	67.1	98	71.5			
No	87	30.7	48	32.9	39	28.5			
COVID-19 altered federal food assistance need	283	100.0	146	100.0	137	100.0	0.513	1	0.474
Yes	218	77.0	115	78.8	103	75.2			
No	65	23.0	31	21.2	34	24.8			
COVID-19 altered emergency food assistance need	270	100.0	142	100.0	128	100.0	0.001	1	0.976
Yes	228	84.4	120	84.5	108	84.4			
No	42	15.6	22	15.5	20	15.6			
COVID-19 altered food access	272	100.0	143	100.0	129	100.0	0.103	1	0.748
Yes	238	87.5	126	88.1	112	86.8			
No	34	12.5	17	11.9	17	13.2			
Use federal food assistance programs in the last 30 days	281	100.0	146	100.0	135	100.0	1.816	1	0.178
Yes	190	67.6	104	71.2	86	63.7			
No	91	32.4	42	28.8	49	36.3			
Aware of emergency food program in their community	283	100.0	146	100.0	137	100.0	0.074	1	0.786
Yes	225	79.5	117	80.1	108	78.8			
No	58	20.5	29	19.9	29	21.2			
Use emergency food assistance for meal delivery services in the last 30 days	283	100.0	146	100.0	137	100.0	0.700	1	0.403
Yes	181	64.0	90	61.6	91	66.4			
No	102	36.0	56	38.4	46	33.6			
Visit programs and senior centers for prepared meals in the last 30 days	283	100.0	146	100.0	137	100.0	0.228	1	0.633
Yes	157	55.5	79	54.1	78	56.9			
No	126	44.5	67	45.9	59	43.1			
Obtain food from a church pantry, food pantry, or food bank in the last 30 days	283	100.0	146	100.0	137	100.0	0.071	1	0.790
Yes	227	80.2	118	80.8	109	79.6			
No	56	19.8	28	19.2	28	20.4			
Visit a soup kitchen or shelter for emergency food in the last 30 days	283	100.0	146	100.0	137	100.0	0.231	1	0.631
Yes	184	65.0	93	63.7	91	66.4			
No	99	35.0	53	36.3	46	33.6			

**Table 6:** The Relationship Between Employment, Food Security Outcomes, and Using Emergency and Federal Food Assistance During COVID-19 in Holyoke, Massachusetts.

Experiences of Food Security, Emergency Food Services, and Federal Food Assistance	Total Sample		Full-Time		Part-Time		Unemployed		χ^2^	*df*	Significance (*p*-Value)
	Number	Percent	Number	Percent	Number	Percent	Number	Percent			
USDA food security category	284	100.0	164	100.0	46	100.0	74	100.0	20.294	4	<0.001 ***
High or Marginal Food Security	76	26.8	57	34.8	9	19.6	10	13.5			
Low Food Security	70	24.6	44	26.8	12	26.1	14	18.9			
Very Low Food Security	138	48.6	63	38.4	25	54.3	50	67.6			
Used federal food assistance programs prior to COVID-19	284	100.0	164	100.0	46	100.0	74	100.0	2.391	2	0.302
Yes	197	69.4	109	66.5	36	78.3	52	70.3			
No	87	30.6	55	33.5	10	21.7	22	29.7			
COVID-19 altered federal food assistance need	284	100.0	164	100.0	46	100.0	74	100.0	8.046	2	0.018 **
Yes	219	77.1	117	71.3	41	89.1	61	82.4			
No	65	22.9	47	28.7	5	10.9	13	17.6			
COVID-19 altered emergency food assistance need	271	100.0	155	100.0	44	100.0	72	100.0	1.819	2	0.403
Yes	229	84.5	128	82.6	40	90.9	61	84.7			
No	42	15.5	27	17.4	4	9.1	11	15.3			
COVID-19 altered food access	273	100.0	155	100.0	45	100.0	73	100.0	1.837	2	0.399
Yes	238	87.2	133	85.8	42	93.3	63	86.3			
No	35	12.8	22	14.2	3	6.7	10	13.7			
Use federal food assistance programs in the last 30 days	282	100.0	162	100.0	46	100.0	74	100.0	6.059	2	0.048 *
Yes	191	67.7	101	62.3	37	80.4	53	71.6			
No	91	32.3	61	37.7	9	19.6	21	28.4			
Aware of emergency food program in their community	284	100.0	164	100.0	46	100.0	74	100.0	1.073	2	0.585
Yes	226	79.6	130	79.3	39	84.8	57	77.0			
No	58	20.4	34	20.7	7	15.2	17	23.0			
Use emergency food assistance for meal delivery services in the last 30 days	284	100.0	164	100.0	46	100.0	74	100.0	1.859	2	0.395
Yes	182	64.1	105	64.0	33	71.7	44	59.5			
No	102	35.9	59	36.0	13	28.3	30	40.5			
Visit programs and senior centers for prepared meals in the last 30 days	284	100.0	164	100.0	46	100.0	74	100.0	1.894	2	0.388
Yes	157	55.3	85	51.8	28	60.9	44	59.5			
No	127	44.7	79	48.2	18	39.1	30	40.5			
Obtain food from a church pantry, food pantry, or food bank in the last 30 days	284	100.0	164	100.0	46	100.0	74	100.0	2.348	2	0.309
Yes	228	80.3	127	77.4	40	87.0	61	82.4			
No	56	19.7	37	22.6	6	13.0	13	17.6			
Visit a soup kitchen or shelter for emergency food in the last 30 days	284	100.0	164	100.0	46	100.0	74	100.0	2.483	2	0.289
Yes	184	64.8	101	61.6	34	73.9	49	66.2			
No			100	35.2	63	38.4	12	26.1	25	33.8	

Significance levels (*p*-values): * α ≤ 0.05, ** α ≤ 0.01, *** α ≤ 0.001.

**Table 7:** The Relationship Between Education, Food Security Outcomes, and Using Emergency and Federal Food Assistance During COVID-19 in Holyoke, Massachusetts.

Experiences of Food Security, Emergency Food Services, and Federal Food Assistance	Total Sample		High School Diploma or GED or Less		Associates or Technical Degree		Bachelors Degree or More		χ^2^	*df*	Significance (*p*-Value)
	Number	Percent	Number	Percent	Number	Percent	Number	Percent			
USDA food security category	284	100.0	76	100.0	129	100.0	79	100.0	29.997	4	<0.001 ***
High or Marginal Food Security	76	26.8	13	17.1	36	27.9	27	34.2			
Low Food Security	70	24.6	9	11.8	31	24.0	30	38.0			
Very Low Food Security	138	48.6	54	71.1	62	48.1	22	27.8			
Used federal food assistance programs prior to COVID-19	284	100.0	76	100.0	129	100.0	79	100.0	15.643	2	<0.001 ***
Yes	197	69.4	66	86.8	84	65.1	47	59.5			
No	87	30.6	10	13.2	45	34.9	32	40.5			
COVID-19 altered federal food assistance need	284	100.0	76	100.0	129	100.0	79	100.0	10.998	2	0.004 **
Yes	219	77.1	69	90.8	93	72.1	57	72.2			
No	65	22.9	7	9.2	36	27.9	22	27.8			
COVID-19 altered emergency food assistance need	271	100.0	74	100.0	120	100.0	77	100.0	13.161	2	<0.001 ***
Yes	229	84.5	72	97.3	94	78.3	63	81.8			
No	42	15.5	2	2.7	26	21.7	14	18.2			
COVID-19 altered food access	273	100.0	75	100.0	120	100.0	78	100.0	4.129	2	0.127
Yes	238	87.2	70	93.3	100	83.3	68	87.2			
No	35	12.8	5	6.7	20	16.7	10	12.8			
Use federal food assistance programs in the last 30 days	282	100.0	75	100.0	128	100.0	79	100.0	12.442	2	0.002 **
Yes	191	67.7	63	84.0	80	62.5	48	60.8			
No	91	32.3	12	16.0	48	37.5	31	39.2			
Aware of emergency food program in their community	284	100.0	76	100.0	129	100.0	79	100.0	3.763	2	0.152
Yes	226	79.6	66	86.8	101	78.3	59	74.7			
No	58	20.4	10	13.2	28	21.7	20	25.3			
Use emergency food assistance for meal delivery services in the last 30 days	284	100.0	76	100.0	129	100.0	79	100.0	7.109	2	0.029 *
Yes	182	64.1	53	69.7	88	68.2	41	51.9			
No	102	35.9	23	30.3	41	31.8	38	48.1			
Visit programs and senior centers for prepared meals in the last 30 days	284	100.0	76	100.0	129	100.0	79	100.0	24.653	2	<0.001 ***
Yes	157	55.3	54	71.1	77	59.7	26	32.9			
No	127	44.7	22	28.9	52	40.3	53	67.1			
Obtain food from a church pantry, food pantry, or food bank in the last 30 days	284	100.0	76	100.0	129	100.0	79	100.0	13.820	2	<0.001 ***
Yes	228	80.3	70	92.1	104	80.6	54	68.4			
No	56	19.7	6	7.9	25	19.4	25	31.6			
Visit a soup kitchen or shelter for emergency food in the last 30 days	284	100.0	76	100.0	129	100.0	79	100.0	13.624	2	<0.001 ***
Yes	184	64.8	60	78.9	84	65.1	40	50.6			
No	100	35.2	16	21.1	45	34.9	39	49.4			

Significance levels (*p*-values): * α ≤ 0.05, ** α ≤ 0.01, *** α ≤ 0.001.

**Table 8:** The Relationship Between Children in the Household, Food Security Outcomes, and Using Emergency and Federal Food Assistance During COVID-19 in Holyoke, Massachusetts.

Experiences of Food Security, Emergency Food Services, and Federal Food Assistance	Total Sample		Children in the Household		No Children in the Household		χ^2^	*df*	Significance (*p*-Value)
	Number	Percent	Number	Percent	Number	Percent			
USDA food security category	284	100.0	224	100.0	60	100.0	7.382	2	0.025 *
High or Marginal Food Security	76	26.8	52	23.2	24	40.0			
Low Food Security	70	24.6	60	26.8	10	16.7			
Very Low Food Security	138	48.6	112	50.0	26	43.3			
Used federal food assistance programs prior to COVID-19	284	100.0	224	100.0	60	100.0	2.122	1	0.145
Yes	197	69.4	160	71.4	37	61.7			
No	87	30.6	64	28.6	23	38.3			
COVID-19 altered federal food assistance need	284	100.0	224	100.0	60	100.0	4.703	1	0.030 *
Yes	219	77.1	179	79.9	40	66.7			
No	65	22.9	45	20.1	20	33.3			
COVID-19 altered emergency food assistance need	271	100.0	212	100.0	59	100.0	19.498	1	<0.001 ***
Yes	229	84.5	190	89.6	39	66.1			
No	42	15.5	22	10.4	20	33.9			
COVID-19 altered food access	273	100.0	214	100.0	59	100.0	1.148	1	0.284
Yes	238	87.2	189	88.3	49	83.1			
No	35	12.8	25	11.7	10	16.9			
Use federal food assistance programs in the last 30 days	282	100.0	223	100.0	59	100.0	3.485	1	0.062
Yes	191	67.7	157	70.4	34	57.6			
No	91	32.3	66	29.6	25	42.4			
Aware of emergency food program in their community	284	100.0	224	100.0	60	100.0	0.981	1	0.322
Yes	226	79.6	181	80.8	45	75.0			
No	58	20.4	43	19.2	15	25.0			
Use emergency food assistance for meal delivery services in the last 30 days	284	100.0	224	100.0	60	100.0	6.556	1	0.010 **
Yes	182	64.1	152	67.9	30	50.0			
No	102	35.9	72	32.1	30	50.0			
Visit programs and senior centers for prepared meals in the last 30 days	284	100.0	224	100.0	60	100.0	0.858	1	0.354
Yes	157	55.3	127	56.7	30	50.0			
No	127	44.7	97	43.3	30	50.0			
Obtain food from a church pantry, food pantry, or food bank in the last 30 days	284	100.0	224	100.0	60	100.0	3.567	1	0.059
Yes	228	80.3	185	82.6	43	71.7			
No	56	19.7	39	17.4	17	28.3			
Visit a soup kitchen or shelter for emergency food in the last 30 days	284	100.0	224	100.0	60	100.0	13.058	1	<0.001 ***
Yes	184	64.8	157	70.1	27	45.0			
No	100	35.2	67	29.9	33	55.0			

Significance levels (*p*-values): * α ≤ 0.05, ** α ≤ 0.01, *** α ≤ 0.001.

**Table 9:** The Relationship Between Household Type, Food Security Outcomes, and Using Emergency and Federal Food Assistance During COVID-19 in Holyoke, Massachusetts.

Experiences of Food Security, Emergency Food Services, and Federal Food Assistance	Total Sample		Single-Parent		Two-Parent		Multifamily		Living with Partner		Living Alone or with Roommates		χ^2^	*df*	Significance (*p*-Value)
	Number	Percent	Number	Percent	Number	Percent	Number	Percent	Number	Percent	Number	Percent			
USDA food security category	284	100.0	35	100.0	70	100.0	50	100.0	89	100.0	40	100.0	17.380	8	0.026 *
High or Marginal Food Security	76	26.8	7	20.0	16	22.9	6	12.0	36	40.4	11	27.5			
Low Food Security	70	24.6	7	20.0	19	27.1	14	28.0	21	23.6	9	22.5			
Very Low Food Security	138	48.6	21	60.0	35	50.0	30	60.0	32	36.0	20	50.0			
Used federal food assistance programs prior to COVID-19	284	100.0	35	100.0	70	100.0	50	100.0	89	100.0	40	100.0	4.396	4	0.355
Yes	197	69.4	23	65.7	46	65.7	39	78.0	58	65.2	31	77.5			
No	87	30.6	12	34.3	24	34.3	11	22.0	31	34.8	9	22.5			
COVID-19 altered federal food assistance need	284	100.0	35	100.0	70	100.0	50	100.0	89	100.0	40	100.0	8.856	4	0.065
Yes	219	77.1	27	77.1	58	82.9	44	88.0	61	68.5	29	72.5			
No	65	22.9	8	22.9	12	17.1	6	12.0	28	31.5	11	27.5			
COVID-19 altered emergency food assistance need	271	100.0	33	100.0	67	100.0	50	100.0	82	100.0	39	100.0	3.241	4	0.518
Yes	229	84.5	28	84.8	58	86.6	45	90.0	68	82.9	30	76.9			
No	42	15.5	5	15.2	9	13.4	5	10.0	14	17.1	9	23.1			
COVID-19 altered food access	273	100.0	34	100.0	68	100.0	50	100.0	82	100.0	39	100.0	6.602	4	0.159
Yes	238	87.2	30	88.2	62	91.2	47	94.0	66	80.5	33	84.6			
No	35	12.8	4	11.8	6	8.8	3	6.0	16	19.5	6	15.4			
Use federal food assistance programs in the last 30 days	282	100.0	34	100.0	70	100.0	50	100.0	88	100.0	40	100.0	6.19	4	0.185
Yes	191	67.7	25	73.5	52	74.3	32	64.0	52	59.1	30	75.0			
No	91	32.3	9	26.5	18	25.7	18	36.0	36	40.9	10	25.0			
Aware of emergency food program in their community	284	100.0	35	100.0	70	100.0	50	100.0	89	100.0	40	100.0	14.606	4	0.006 **
Yes	226	79.6	32	91.4	60	85.7	33	66.0	74	83.1	27	67.5			
No	58	20.4	3	8.6	10	14.3	17	34.0	15	16.9	13	32.5			
Use emergency food assistance for meal delivery services in the last 30 days	284	100.0	35	100.0	70	100.0	50	100.0	89	100.0	40	100.0	7.564	4	0.109
Yes	182	64.1	18	51.4	52	74.3	35	70.0	54	60.7	23	57.5			
No	102	35.9	17	48.6	18	25.7	15	30.0	35	39.3	17	42.5			
Visit programs and senior centers for prepared meals in the last 30 days	284	100.0	35	100.0	70	100.0	50	100.0	89	100.0	40	100.0	10.265	4	0.036 *
Yes	157	55.3	20	57.1	43	61.4	32	64.0	37	41.6	25	62.5			
No	127	44.7	15	42.9	27	38.6	18	36.0	52	58.4	15	37.5			
Obtain food from a church pantry, food pantry, or food bank in the last 30 days	284	100.0	35	100.0	70	100.0	50	100.0	89	100.0	40	100.0	3.451	4	0.485
Yes	228	80.3	26	74.3	57	81.4	42	84.0	68	76.4	35	87.5			
No	56	19.7	9	25.7	13	18.6	8	16.0	21	23.6	5	12.5			
Visit a soup kitchen or shelter for emergency food in the last 30 days	284	100.0	35	100.0	70	100.0	50	100.0	89	100.0	40	100.0	7.224	4	0.125
Yes	184	64.8	22	62.9	52	74.3	36	72.0	52	58.4	22	55.0			
No	100	35.2	13	37.1	18	25.7	14	28.0	37	41.6	18	45.0			

Significance levels (*p*-values): * α ≤ 0.05, ** α ≤ 0.01.

**Table 10:** A Binary Logistic Regression Analysis for Using Federal Food Assistance Programs Prior to COVID-19.

	Used Federal Food Assistance Programs Prior to COVID-19					
	β Coefficient	Standard Error (SE)	Odds Ratio (OR)	95% Confidence Interval (CI)		*p*-Value
				Lower	Upper	
Age						
30–39 years old	–0.371	0.354	0.690	0.345	1.381	0.690
40 years or older	–0.714	0.351	0.490	0.246	0.975	0.042 *
Income						
USD 50,000 or more	–0.113	0.315	0.893	0.482	1.654	0.719
Hispanic/Latino heritage						
White	–0.689	0.290	0.502	0.285	0.886	0.018 *
Gender						
Men	0.339	0.310	1.403	0.764	2.578	0.275
Employment						
Part-time	0.092	0.356	1.097	0.546	2.205	0.795
Unemployed	–0.173	0.477	0.841	0.330	2.143	0.717
Education						
Some college, Associate, or Technical Degree	–1.394	0.455	0.248	0.102	0.605	0.002 **
Bachelors Degree or more	–0.285	0.321	0.752	0.401	1.411	0.375
Children in the household						
None	–0.508	0.387	0.602	0.282	1.285	0.189
Household type						
Two-parent	0.794	0.611	2.212	0.669	7.321	0.193
Multifamily	0.905	0.578	2.473	0.797	7.671	0.117
Living with partner	0.146	0.615	1.158	0.347	3.864	0.812
Living with roommates or alone	0.801	0.555	2.227	0.751	6.605	0.149

Notes: The reference group for age is 18–29 years old. The reference group for income is $0–49,999. The reference group for Hispanic/Latino heritage is Puerto Rican. The reference group for gender is women. The reference group for employment is full-time. The reference group for education is high school diploma or GED and less. The reference group for children in the household is households with children. The reference group for household type is single parent. Significance levels (*p*-values): * α ≤ 0.05, ** α ≤ 0.01.

**Table 11:** A Binary Logistic Regression Analysis for the Impact of COVID-19 Pandemic on Federal Food Assistance Need.

	COVID-19 Altered Federal Food Assistance Need					
	β Coefficient	Standard Error (SE)	Odds Ratio (OR)	95% Confidence Interval (CI)		*p*-Value
				Lower	Upper	
Age						
30–39 years old	–1.789	0.466	0.167	0.067	0.416	<0.001 ***
40 years or older	–1.250	0.390	0.286	0.133	0.615	<0.001 ***
Income						
USD 50,000 or more	–0.746	0.361	0.474	0.233	0.963	0.039 *
Hispanic/Latino heritage						
White	–0.213	0.326	0.809	0.427	1.530	0.514
Gender						
Men	0.197	0.358	1.217	0.603	2.456	0.583
Employment						
Part-time	0.498	0.424	1.645	0.717	3.775	0.240
Unemployed	–0.538	0.628	0.584	0.170	2.000	0.392
Education						
Some college, Associate, or Technical Degree	–0.747	0.533	0.474	0.167	1.346	0.161
Bachelors Degree or more	0.118	0.361	1.125	0.554	2.285	0.745
Children in the household						
None	–0.425	0.414	0.654	0.290	1.471	0.304
Household type						
Two-parent	–0.410	0.645	0.663	0.188	2.347	0.525
Multifamily	–1.169	0.629	0.311	0.091	1.067	0.063
Living with partner	−1.512	0.708	0.220	0.055	0.882	0.033 *
Living with roommates or alone	–0.314	0.560	0.731	0.244	2.190	0.575

Notes: The reference group for age is 18–29 years old. The reference group for income is $0–49,999. The reference group for Hispanic/Latino heritage is Puerto Rican. The reference group for gender is women. The reference group for employment is full-time. The reference group for education is high school diploma or GED and less. The reference group for children in the household is households with children. The reference group for household type is single-parent. Significance levels (*p*-values): * α ≤ 0.05, *** α ≤ 0.001.

**Table 12:** A Binary Logistic Regression Analysis for the Impact of the COVID-19 Pandemic on Emergency Food Assistance Need.

	COVID-19 Altered Emergency Food Assistance Need					
	β Coefficient	Standard Error (SE)	Odds Ratio (OR)	95% Confidence Interval (CI)		*p*-Value
				Lower	Upper	
Age						
30–39 years old	–0.812	0.496	0.444	0.168	1.173	0.101
40 years or older	–0.658	0.465	0.518	0.208	1.289	0.157
Income						
USD 50,000 or more	–0.646	0.425	0.524	0.228	1.206	0.129
Hispanic/Latino heritage						
White	–0.179	0.385	0.836	0.393	1.780	0.642
Gender						
Man	0.261	0.405	1.298	0.588	2.869	0.519
Employment						
Part-time	0.157	0.477	1.170	0.459	2.982	0.743
Unemployed	–0.367	0.684	0.693	0.181	2.648	0.591
Education						
Some college, Associate, or Technical Degree	–1.729	0.817	0.177	0.036	0.880	0.034 *
Bachelors Degree or more	0.368	0.412	1.445	0.645	3.240	0.371
Children in the household						
None	–1.342	0.445	0.261	0.109	0.625	0.003 **
Household type						
Two-parent	−0.188	0.725	0.828	0.200	3.432	0.795
Multifamily	–0.455	0.695	0.635	0.162	2.479	0.513
Living with partner	–0.674	0.757	0.510	0.116	2.249	0.374
Living with roommates or alone	–0.267	0.623	0.765	0.226	2.595	0.668

Notes: The reference group for age is 18–29 years old. The reference group for income is USD 0–49,999. The reference group for Hispanic/Latino heritage is Puerto Rican. The reference group for gender is women. The reference group for employment is full time. The reference group for education is high school diploma or GED and less. The reference group for children in the household is households with children. The reference group for household type is single-parent. Significance levels (*p*-values): * α ≤ 0.05, ** α ≤ 0.01.

**Table 13:** A Binary Logistic Regression Analysis for the Impact of the COVID-19 Pandemic on Food Access.

	COVID-19 Altered Food Access					
	β Coefficient	Standard Error (SE)	Odds Ratio (OR)	95% Confidence Interval (CI)		*p*-Value
				Lower	Upper	
Age						
30–39 years old	–0.872	0.495	0.418	0.159	1.103	0.078
40 years or older	–1.313	0.518	0.269	0.098	0.742	0.011 **
Income						
USD 50,000 or more	–0.597	0.447	0.551	0.229	1.321	0.181
Hispanic/Latino heritage						
White	–0.126	0.411	0.882	0.394	1.974	0.760
Gender						
Man	0.331	0.434	1.393	0.595	3.263	0.445
Employment						
Part-time	0.087	0.487	1.091	0.420	2.831	0.858
Unemployed	–0.547	0.731	0.579	0.138	2.424	0.454
Education						
Some college, Associates, or Technical Degree	–0.103	0.658	0.902	0.249	3.275	0.876
Bachelors Degree or more	0.531	0.460	1.701	0.690	4.194	0.248
Children in the household						
None	–0.043	0.504	0.958	0.357	2.573	0.933
Household type						
Two-parent	–0.436	0.799	0.647	0.135	3.096	0.585
Multifamily	–0.905	0.766	0.405	0.090	1.815	0.237
Living with partner	–1.394	0.886	0.248	0.044	1.410	0.116
Living with roommates or alone	0.405	0.685	1.499	0.392	5.736	0.555

Notes: The reference group for age is 18–29 years old. The reference group for income is USD 0–49,999. The reference group for Hispanic/Latino heritage is Puerto Rican. The reference group for gender is women. The reference group for employment is full- time. The reference group for education is high school diploma or GED and less. The reference group for children in the household is households with children. The reference group for household type is single-parent. Significance levels (*p*-values): ** α ≤ 0.01.

**Table 14:** A Binary Logistic Regression Analysis for Using Federal Food Assistance Programs.

	Use Federal Food Assistance Programs in the Last 30 Days					
	β Coefficient	Standard Error (SE)	Odds Ratio (OR)	95% Confidence Interval (CI)		*p*-Value
				Lower	Upper	
Age						
30–39 years old	–0.365	0.352	0.694	0.348	1.383	0.300
40 years or older	–0.211	0.340	0.810	0.416	1.577	0.534
Income						
USD 50,000 or more	–0.542	0.302	0.582	0.322	1.052	0.073
Hispanic/Latino heritage						
White	–0.204	0.284	0.815	0.468	1.422	0.472
Gender						
Man	–0.215	0.301	0.807	0.447	1.456	0.476
Employment						
Part-time	0.134	0.348	1.143	0.578	2.259	0.701
Unemployed	−0.410	0.483	0.664	0.258	1.710	0.396
Education						
Some college, Associates, or Technical Degree	−0.794	0.434	0.452	0.193	1.059	0.068
Bachelors Degree or more	−0.059	0.319	0.943	0.504	1.763	0.853
Children in the household						
None	−0.811	0.384	0.444	0.209	0.943	0.035 *
Household type						
Two-parent	0.635	0.616	1.887	0.564	6.305	0.302
Multifamily	0.298	0.566	1.347	0.445	4.082	0.598
Living with partner	0.965	0.572	2.625	0.856	8.050	0.091
Living with roommates or alone	0.727	0.530	2.070	0.733	5.843	0.170

Notes: The reference group for age is 18–29 years old. The reference group for income is USD 0–49,999. The reference group for Hispanic/Latino heritage is Puerto Rican. The reference group for gender is women. The reference group for employment is full-time. The reference group for education is high school diploma or GED and less. The reference group for children in the household is households with children. The reference group for household type is single-parent. Significance levels (*p*-values): * α ≤ 0.05.

**Table 15:** A Binary Logistic Regression Analysis for the Awareness of Emergency Food Programs in Community.

	Aware of Emergency Food Programs in Their Community					
	β Coefficient	Standard Error (SE)	Odds Ratio (OR)	95% Confidence Interval (CI)		*p*-Value
				Lower	Upper	
Age						
30–39 years old	0.446	0.411	1.562	0.698	3.497	0.278
40 years or older	0.231	0.416	1.260	0.558	2.848	0.578
Income						
USD 50,000 or more	−0.671	0.359	0.511	0.253	1.032	0.061
Hispanic/Latino heritage						
White	0.677	0.351	1.968	0.989	3.918	0.054
Gender						
Men	−0.014	0.344	0.986	0.502	1.935	0.966
Employment						
Part-time	−0.312	0.394	0.732	0.338	1.586	0.429
Unemployed	−0.654	0.543	0.520	0.179	1.507	0.228
Education						
Some college, Associate, or Technical Degree	−1.068	0.494	0.344	0.131	0.905	0.031 *
Bachelors Degree or more	−0.492	0.378	0.611	0.291	1.283	0.193
Children in the household						
None	−0.029	0.438	0.972	0.412	2.292	0.948
Household type						
Two-parent	−1.681	0.748	0.186	0.043	0.806	0.025 *
Multifamily	−1.387	0.586	0.250	0.079	0.787	0.018 *
Living with partner	0.050	0.551	1.052	0.357	3.100	0.927
Living with roommates or alone	−1.322	0.557	0.267	0.090	0.794	0.018 *

Notes: The reference group for age is 18–29 years old. The reference group for income is USD 0–49,999. The reference group for Hispanic/Latino heritage is Puerto Rican. The reference group for gender is women. The reference group for employment is full-time. The reference group for education is high school diploma or GED and less. The reference group for children in the household is households with children. The reference group for household type is single-parent. Significance levels (*p*-values): * α ≤ 0.05.

**Table 16:** A Binary Logistic Regression Analysis for Using Emergency Meal Delivery Services.

	Use Emergency Food Assistance for Meal Delivery Services in the Last 30 Days					
	β Coefficient	Standard Error (SE)	Odds Ratio (OR)	95% Confidence Interval (CI)		*p*-Value
				Lower	Upper	
Age						
30–39 years old	−0.362	0.339	0.696	0.359	1.353	0.286
40 years or older	0.139	0.329	1.149	0.602	2.191	0.673
Income						
USD 50,000 or more	0.122	0.301	1.130	0.627	2.037	0.684
Hispanic/Latino heritage						
White	0.281	0.283	1.324	0.760	2.308	0.322
Gender						
Man	0.255	0.291	1.290	0.730	2.282	0.381
Employment						
Part-time	−0.170	0.329	0.844	0.443	1.606	0.605
Unemployed	−0.568	0.428	0.566	0.245	1.312	0.185
Education						
Some college, Associate, or Technical Degree	−0.686	0.388	0.503	0.235	1.077	0.077
Bachelors Degree or more	−0.630	0.316	0.532	0.287	0.989	0.046 *
Children in the household						
None	−0.723	0.363	0.485	0.238	0.988	0.046 *
Household type						
Two parent	0.490	0.533	1.633	0.575	4.639	0.357
Multifamily	−0.424	0.509	0.654	0.241	1.775	0.405
Living with partner	−0.144	0.526	0.866	0.309	2.427	0.785
Living with roommates or alone	0.038	0.482	1.039	0.404	2.672	0.937

Notes: The reference group for age is 18–29 years old. The reference group for income is USD 0–49,999. The reference group for Hispanic/Latino heritage is Puerto Rican. The reference group for gender is women. The reference group for employment is full-time. The reference group for education is high school diploma or GED and less. The reference group for children in the household is households with children. The reference group for household type is single-parent. Significance levels (*p*-values): * α ≤ 0.05.

**Table 17:** A Binary Logistic Regression Analysis for Visiting Programs and Senior Centers for Prepared Meals.

	Visit Programs and Senior Centers for Prepared Meals in the Last 30 Days					
	β Coefficient	Standard Error (SE)	Odds Ratio (OR)	95% Confidence Interval (CI)		*p*-Value
				Lower	Upper	
Age						
30–39 years old	−0.722	0.333	0.486	0.253	0.933	0.030 *
40 years or older	−0.273	0.326	0.761	0.402	1.441	0.402
Income						
USD 50,000 or more	−0.368	0.296	0.692	0.387	1.237	0.214
Hispanic/Latino heritage						
White	−0.253	0.279	0.776	0.449	1.341	0.364
Gender						
Man	0.458	0.293	1.581	0.890	2.807	0.118
Employment						
Part-time	0.067	0.334	1.069	0.556	2.056	0.841
Unemployed	0.064	0.421	1.066	0.467	2.432	0.879
Education						
Some college, Associate, or Technical Degree	−1.170	0.388	0.310	0.145	0.664	0.003 **
Bachelors Degree or more	−0.978	0.319	0.376	0.201	0.702	0.002 **
Children in the household						
None	−0.250	0.373	0.779	0.375	1.619	0.503
Household type						
Two-parent	0.334	0.549	1.396	0.476	4.094	0.543
Multifamily	0.101	0.516	1.106	0.403	3.040	0.845
Living with partner	0.150	0.537	1.162	0.406	3.326	0.780
Living with roommates or alone	0.867	0.499	2.380	0.894	6.336	0.082

Notes: The reference group for age is 18–29 years old. The reference group for income is USD 0–49,999. The reference group for Hispanic/Latino heritage is Puerto Rican. The reference group for gender is women. The reference group for employment is full-time. The reference group for education is high school diploma or GED and less. The reference group for children in the household is households with children. The reference group for household type is single-parent. Significance levels (*p*-values): * α ≤ 0.05, ** α ≤ 0.01.

**Table 18:** A Binary Logistic Regression Analysis for Obtaining Food from a Church Pantry, Food Pantry, or Food Bank.

	Obtain Food from a Church Pantry, Food Pantry, or Food Bank in the Last 30 Days					
	β Coefficient	Standard Error (SE)	Odds Ratio (OR)	95% Confidence Interval (CI)		*p*-Value
				Lower	Upper	
Age						
30–39 years old	−0.565	0.432	0.568	0.244	1.325	0.191
40 years or older	−0.069	0.383	0.933	0.441	1.975	0.857
Income						
USD 50,000 or more	0.178	0.359	1.195	0.590	2.416	0.621
Hispanic/Latino heritage						
White	−0.110	0.330	0.896	0.469	1.712	0.739
Gender						
Man	−0.105	0.353	0.900	0.451	1.799	0.766
Employment						
Part-time	0.167	0.407	1.181	0.532	2.625	0.682
Unemployed	−0.298	0.568	0.742	0.244	2.259	0.600
Education						
Some college, Associate, or Technical Degree	−1.528	0.533	0.217	0.076	0.617	0.004 **
Bachelors Degree or more	−0.664	0.355	0.515	0.257	1.031	0.061
Children in the household						
None	−0.934	0.417	0.393	0.173	0.890	0.025 *
Household type						
Two-parent	1.609	0.718	4.999	1.223	20.422	0.025 *
Multifamily	1.157	0.706	3.180	0.798	12.679	0.101
Living with partner	1.058	0.732	2.882	0.687	12.098	0.148
Living with roommates or alone	0.998	0.657	2.712	0.749	9.823	0.129

Notes: The reference group for age is 18–29 years old. The reference group for income is USD 0–49,999. The reference group for Hispanic/Latino heritage is Puerto Rican. The reference group for gender is women. The reference group for employment is full-time. The reference group for education is high school diploma or GED and less. The reference group for children in the household is households with children. The reference group for household type is single-parent. Significance levels (*p*-values): * α ≤ 0.05, ** α ≤ 0.01.

**Table 19:** A Binary Logistic Regression Analysis for Visiting a Soup Kitchen or Shelter.

	Visit A Soup Kitchen Or Shelter For Emergency Food In The Last 30 Days					
	β Coefficient	Standard Error (SE)	Odds Ratio (OR)	95% Confidence Interval (CI)		*p*-Value
				Lower	Upper	
Age						
30–39 years old	−0.421	0.348	0.656	0.332	1.299	0.227
40 years or older	−0.277	0.339	0.758	0.390	1.473	0.413
Income						
USD 50,000 or more	−0.783	0.305	0.457	0.251	0.831	0.010 **
Hispanic/Latino heritage						
White	0.081	0.286	1.084	0.619	1.898	0.778
Gender						
Man	0.443	0.303	1.558	0.860	2.821	0.143
Employment						
Part-time	0.095	0.345	1.099	0.559	2.162	0.784
Unemployed	−0.236	0.456	0.790	0.323	1.929	0.604
Education						
Some college, Associate, or Technical Degree	−0.921	0.408	0.398	0.179	0.886	0.024 *
Bachelors Degree or more	−0.483	0.317	0.617	0.331	1.149	0.128
Children in the household						
None	−0.860	0.366	0.423	0.206	0.867	0.019 *
Household type						
Two-parent	−0.024	0.544	0.976	0.336	2.832	0.964
Multifamily	−0.781	0.524	0.458	0.164	1.280	0.137
Living with partner	−0.393	0.542	0.675	0.233	1.954	0.469
Living with roommates or alone	−0.015	0.492	0.985	0.376	2.582	0.976

Notes: The reference group for age is 18–29 years old. The reference group for income is USD 0–49,999. The reference group for Hispanic/Latino heritage is Puerto Rican. The reference group for gender is women. The reference group for employment is full-time. The reference group for education is high school diploma or GED and less. The reference group for children in the household is households with children. The reference group for household type is single parent. Significance levels (*p*-values): * α ≤ 0.05, ** α ≤ 0.01.

**Table 20:** Multinomial Regression of Food Security Categories by Demographic and Household Characteristics.

Demographic and Household Characteristics	Food Security Category											
	Low Food Security						Very Low Food Security					
	β Coefficient	Standard Error (SE)	Relative Risk Ratio (RRR)	95% Confidence Interval (CI)		*p*-Value	β Coefficient	Standard Error (SE)	Relative Risk Ratio (RRR)	95% Confidence Interval (CI)		*p*-Value
				Lower	Upper					Lower	Upper	
Age												
18–29 Years Old	0.205	0.489	1.228	0.471	3.205	0.675	1.095	0.425	2.989	1.299	6.877	0.009 **
30–39 Years Old	0.599	0.434	1.821	0.778	4.262	0.167	1.050	0.422	2.856	1.248	6.535	0.012 *
Income												
USD 0–49,999	0.433	0.393	1.541	0.713	3.332	0.271	1.018	0.368	2.768	1.346	5.689	0.006 **
Hispanic/Latino heritage												
Puerto Rican	−0.043	0.371	0.958	0.463	1.984	0.909	0.236	0.349	1.266	0.639	2.508	0.499
Gender												
Women	−0.668	0.408	0.513	0.231	1.139	0.101	−0.505	0.365	0.604	0.295	1.235	0.167
Employment												
Full-time	−0.864	0.527	0.422	0.150	1.184	0.101	−1.446	0.463	0.235	0.095	0.584	<0.001 ***
Part-time	−0.335	0.654	0.715	0.199	2.576	0.608	−0.998	0.583	0.369	0.118	1.155	0.087
Education												
High School Diploma or GED or less	−1.130	0.574	0.323	0.105	0.994	0.049 *	0.842	0.496	2.321	0.878	6.138	0.090
Associates or technical degree and some college or less	−0.481	0.401	0.618	0.281	1.358	0.231	0.543	0.405	1.721	0.778	3.806	0.180
Children in Household												
Yes	1.389	0.538	4.010	1.398	11.500	0.009 **	0.606	0.444	1.833	0.767	4.378	0.173
Household Type												
Single Parent	−0.022	0.804	0.979	0.203	4.727	0.978	0.506	0.680	1.658	0.438	6.282	0.457
Two Parent	−0.005	0.712	0.995	0.247	4.017	0.995	0.466	0.632	1.594	0.462	5.501	0.461
Multifamily	0.675	0.796	1.965	0.413	9.347	0.396	1.090	0.721	2.974	0.724	12.216	0.131
Living with Partner	−1.261	0.681	0.283	0.075	1.077	0.064	−0.577	0.596	0.561	0.175	1.806	0.333

Notes: The reference group for the food security category is participants who screened as having high or marginal food security. The reference group for age is 40 years or older. The reference group for income is USD 50,000 or more. The reference group for Hispanic/Latino heritage is White. The reference group for gender is men. The reference group for employment is unemployed. The reference group for education is Bachelors degree or higher. The reference group for children in the household is none. The reference group for household type is living with roommates or alone. Significance levels (*p*-values): * α ≤ 0.05, ** α ≤ 0.01, *** α ≤ 0.001.

**Table 21:** Hierarchical Regression of Food Security by Demographic, Socioeconomic, and Household Characteristics.

	Model 1						Model 2						Model 3					
	β Coefficient	Standard Error	*p*-Value	R^2^	ΔR^2^	ΔF	β Coefficient	Standard Error	*p*-Value	R^2^	ΔR^2^	ΔF	β Coefficient	Standard Error	*p*-Value	R^2^	ΔR^2^	ΔF
Demographic Characteristics				0.054	0.054	5.286												
Age	−0.208	0.062	<0.001 ***				−0.212	0.061	<0.001 ***				−0.171	0.058	0.004 **			
Gender	−0.113	0.098	0.249				−0.057	0.103	0.583				0.073	0.099	0.464			
Hispanic/Latino heritage	−0.147	0.103	0.156				−0.114	0.104	0.275				−0.056	0.098	0.571			
Household Characteristics										0.070	0.016	2.375						
Household Type							−0.064	0.042	0.129				−0.061	0.040	0.131			
Children in Household							−0.139	0.125	0.269				−0.143	0.117	0.228			
Socioeconomic Characteristics																0.191	0.121	13.689
Education													−0.164	0.066	0.014 *			
Income													−0.301	0.101	0.003 **			
Employment													−0.188	0.058	<0.001 ***			

Notes: Significance levels (*p*-values): * α ≤ 0.05, ** α ≤ 0.01, *** α ≤ 0.001.

## Data Availability

The original contributions presented in the study are included in the article; further inquiries can be directed to the corresponding author. The data are not publicly available because respondents were promised anonymity and confidentiality for their participation in the study.

## Supplementary Materials

The following supporting information can be downloaded at: https://www.mdpi.com/article/10.3390/nu16213666/s1, Figure S1: Map of Holyoke, Massachusetts, depicting categories of emergency food assistance programs; Table S1: Dependent and Independent Variable Labels and Codes; Table S2: Sample characteristics.
