# Supplementary material for: Predictors of Food Insecurity and Food Assistance Program Usage Among Puerto Ricans Before and During the COVID-19 Pandemic in Holyoke, Massachusetts
Source: Nutrients. 2024 Oct 28;16(21):3666. doi: 10.3390/nu16213666 (PMC11547546; doi:10.3390/nu16213666)
Supplement: Supplementary file 1 [file nutrients-16-03666-s001.zip › nutrients-3247318-supplementary.pdf]

Supplementary Materials

S1

The study identified 44 emergency food outlets in Holyoke, Massachusetts, in 2022. This study explores seven types of emergency food outlets: community fridges, food banks, food delivery services, food drives, food pantries, soup kitchens, and community gardens. Holyoke's most prevalent emergency food outlet was community gardens (31.8%). There were 11 food banks (25.0%), 11 food pantries (25.0%), three food deliveries (6.8%), three food drives (6.8%), and two soup kitchens (4.5%) (See Figure S1). Figure S1 also includes an inset to zoom in and locate Holyoke's downtown region, where many emergency food outlets operate. For more information on collecting this data to uncover the available emergency food outlets in Holyoke and mapping the availability of these resources, please refer to [164].

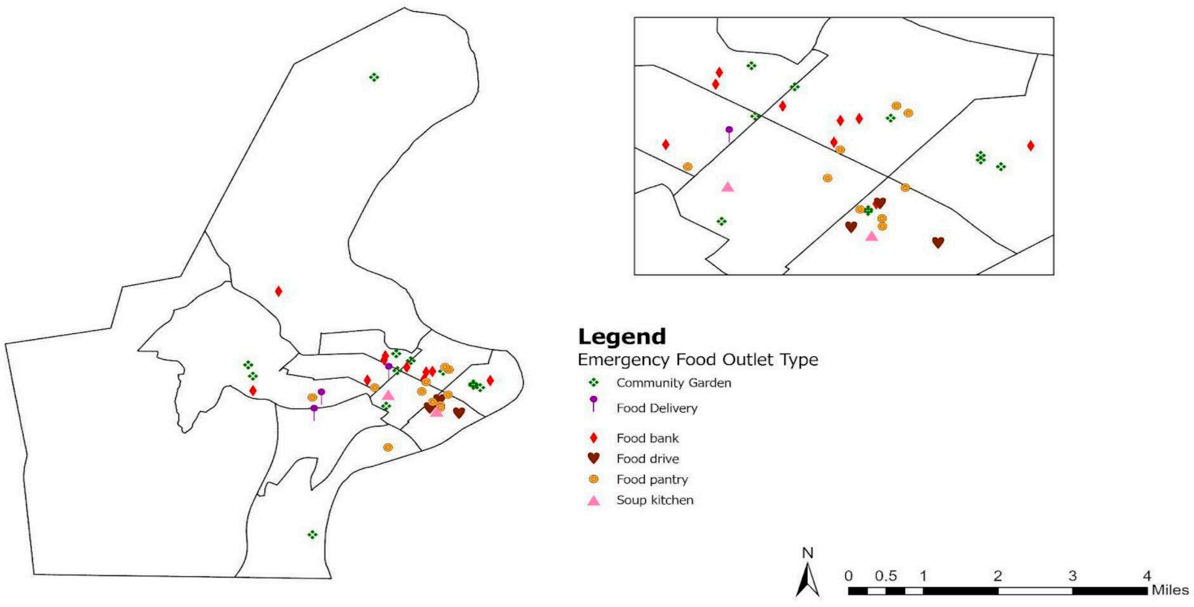

Figure S1. Map of Holyoke, Massachusetts, depicting categories of emergency food assistance programs.

S2

The independent variables were demographic and household characteristics of interest, including Hispanic/Latino heritage[1], gender[2], age, employment, educational attainment, children in the household, household type, and household income. All the independent variables were categorical.

The dependent variables were food security status measured by the USDA Six Item Short Form of the Food Security Module and self-reported food access needs and assistance participation. Ten of the dependent variables were binary. The food security category was a multinomial dependent variable. Food assistance participation includes the use of federal food benefits and various emergency food assistance programs such as pantries, soup kitchens, and food banks before the COVID-19 pandemic and within the last 30 days of when the respondent took the survey in 2022. Table S1 outlines these variables, highlighting the codes for all the variables.

Table S1. Dependent and Independent Variable Labels and Codes.

Dependent Variables	Abbreviated Labels	Numerical Codes	Independent Variables	Abbreviated Labels	Numerical Codes
Used federal food assistance programs prior to COVID	UFF		Gender identification	GI	
Yes		1	Woman		1
No		0	Man		2
Household needed federal food assistance because of COVID	NFF		Age identification	AI	
Yes		1	18-29 Years old		1
No		0	30-39 Years old		2
Household needed emergency food assistance because of COVID	NEF		40 Years or older		3
Yes		1	Employment	EM	
No		0	Full-time		1
Used federal food assistance programs in past 30 days	FFU		Part-time		2
Yes		1	Unemployed		3
No		0	Education	ED	
COVID altered food access	AFA		High school diploma or GED or less		1
Yes		1	Associates degree or some college or less		2
No		0	Bachelors degree or higher		3
Aware of emergency food program in community	EFA		Children in the household	CH	
Yes		1	Yes		1
No		0	No		2
Used emergency food assistance for meal delivery services in past 30 days	MDS		Household type	HT	
Yes		1	Single parent		1
No		0	Two parents		2
Visited programs and senior centers for prepared meals in the past 30 days	VPS		Multifamily		3
Yes		1	Living alone or with roommates		4
No		0	Household income	HI	
Obtained food from church pantry, food pantry, or food bank in the past 12 months	CPF		$0-$49,999		1
Yes		1	$50,000-99,999		2
No		0	Hispanic/Latino heritage	PR	
Visited a soup kitchen or shelter for emergency food in the past 12 months	SKS		Puerto Rican		1
Yes		1	Non-Hispanic/Latino White		2
No		0			
USDA Food Security Category	FSC				
High or marginal food security		1			
Low food security		2			
Very low food security		3			

[1] Due to the predominant presence of Puerto Ricans in the Holyoke sample, we dichotomized the sample into two categories: Puerto Rican or non-Hispanic/Latino White. Puerto Ricans hold U.S. citizenship, and Puerto Rico is classified as a U.S. territory. Thus, we do not categorize this group by nationality or country of origin. Instead, we identify Puerto Ricans by their Hispanic/Latino heritage, acknowledging the unique cultural, social, and historical experiences that distinguish them within the broader context of U.S. citizens. This approach allows a more nuanced understanding of the population's identity beyond mere geopolitical definitions.

[2] Although we concentrated efforts on implementing gender-diverse options on the survey to avoid perpetuating exclusionary binary gender and heteronormative ideologies, we received only four responses identifying as non-binary. Due to this relatively low count, we omitted these surveys from the statistical analysis to avoid potential errors.

S3

Table S2 depicts the demographic and household characteristics. Most of the respondents were Puerto Rican (64.8%), women (51.6%), worked full-time (57.7%), lived in households earning $0-49,999 (60.9%), and had children in their household (78.9%). The age distribution varied: 31.7% were 18-29, 35.9% were 30-39, and 32.4% were 40 or older. Regarding educational attainment, more than a quarter of the sample did not go to college—26.8% had a high school diploma or GED or less. The remainder of the sample had an associate or technical degree and some college or less (45.4%) or acquired a bachelor's degree or higher (27.8%). The composition of households was heterogeneous: 12.3% of respondents were from single-parent households, 24.6% from two-parent households, 17.6% from multi-family households, 31.3% lived with a partner, and 14.1% lived alone or with roommates.

Table S2. Sample characteristics.

Characteristics	Total Sample	
	Number	Percent
**Hispanic/Latino Heritage**	**284**	**100.0**
Puerto Rican	184	64.8
Non-Hispanic/Latino White	100	35.2
**Gender identification**	**283**	**100.0**
Woman	146	51.6
Man	137	48.4
**Age**	**284**	**100.0**
18-29 years old	90	31.7
30-39 years old	102	35.9
40 years or older	92	32.4
**Employment**	**284**	**100.0**
Working full-time	164	57.7
Working part-time	46	16.2
Unemployed	74	26.1
**Educational attainment**	**284**	**100.0**
High school diploma or GED or less	76	26.8
Associate or technical degree and some college or less	129	45.4
Bachelor's degree and/or graduate degree	79	27.8
**Household income**	**284**	**100.0**
$0 - $49,999	173	60.9
$50,000 or more	111	39.1
**Household type**	**284**	**100.0**
Single Parent	35	12.3
Two Parent	70	24.6
Multi-family	50	17.6
Living with partner	89	31.3
Living alone or with roommates	40	14.1
**Children (under the age of 18) in the household**	**284**	**100.0**
Yes	224	78.9
No	60	21.1
